# A Meta-Analysis of the Protein Components in Rattlesnake Venom

**DOI:** 10.3390/toxins13060372

**Published:** 2021-05-23

**Authors:** Anant Deshwal, Phuc Phan, Jyotishka Datta, Ragupathy Kannan, Suresh Kumar Thallapuranam

**Affiliations:** 1 Division of Biology, University of Tennessee, Knoxville, TN 37996, USA; adeshwal@utk.edu; 2 Department of Chemistry and Biochemistry, University of Arkansas, Fayetteville, AR 72701, USA; phucphan@uark.edu; 3 Department of Statistics, Virginia Polytechnic Institute and State University, Blacksburg, VA 24061, USA; jyotishka@vt.edu; 4 Department of Biology, University of Arkansas-Fort Smith, Fort Smith, AR 72913, USA; Ragupathy.Kannan@uafs.edu

**Keywords:** rattlesnake, *Crotalus*, *Sistrurus*, venom, toxin, association

## Abstract

The specificity and potency of venom components give them a unique advantage in developing various pharmaceutical drugs. Though venom is a cocktail of proteins, rarely are the synergy and association between various venom components studied. Understanding the relationship between various components of venom is critical in medical research. Using meta-analysis, we observed underlying patterns and associations in the appearance of the toxin families. For *Crotalus*, Dis has the most associations with the following toxins: PDE; BPP; CRL; CRiSP; LAAO; SVMP P-I and LAAO; SVMP P-III and LAAO. In *Sistrurus* venom, CTL and NGF have the most associations. These associations can predict the presence of proteins in novel venom and understand synergies between venom components for enhanced bioactivity. Using this approach, the need to revisit the classification of proteins as major components or minor components is highlighted. The revised classification of venom components is based on ubiquity, bioactivity, the number of associations, and synergies. The revised classification can be expected to trigger increased research on venom components, such as NGF, which have high biomedical significance. Using hierarchical clustering, we observed that the genera’s venom compositions were similar, based on functional characteristics rather than phylogenetic relationships.

## 1. Introduction

Venom study has become an integral part of the biomedical research [1] as various venom components have been critical in the development of new pharmaceutical drugs [2] that are potentially useful for the treatment of diabetes, strokes, heart attacks [3,4], and cancer [5,6,7,8,9,10,11,12]. For most extant research, venom is sourced from various venomous organisms, such as snakes, scorpions, spiders, etc. Among snakes, venomous snakes are distributed mainly in three families: Atractaspidae, Elapidae, Viperidae [13]. Venom from these snake families is highly complex and variable in composition [14,15,16]. The variation in biochemical composition of snake venom can occur between closely related species and within a single species itself [1,17,18,19,20,21,22]. For example, intragenus or intraspecific variation in venom in pit vipers and adders [17,23] has been correlated to diet [17,18,24,25] or topographical features [26,27]. One of the primary reasons for high diversity and plasticity in snake venom is frequent duplication of toxin-encoding genes and recruitment strategies [28,29,30,31,32] followed by functional and structural diversification [1,33,34,35,36,37].

Within the North and South American continent, it is suggested that the venom of Crotalidae has the highest variation in toxicity is associated with high proteolytic activity [38]. Rattlesnakes are a part of the Crotalinae subfamily and consist of two genera *Crotalus* and *Sistrurus.* They are native to the Americas ranging from southern Alberta, Saskatchewan, and southern British Columbia in Canada to central Argentina. There are approximately 32 species of rattlesnakes within the *Crotalus* and *Sistrurus* genus [39]. These snakes are found in many habitat types ranging from the Sonoran Desert of northwestern Mexico to alpine and cloud forest in central and southern Mexico [39]. They occur from below sea level in desert basins in California to about 4500 m in the Transverse Volcanic Cordillera of central Mexico [39]. Mexican Plateau and its fringing mountains have the highest diversity of rattlesnakes [39]. This high variability in the habitat type, altitude, and associated diet types, along with a large geographical range, allows the rattlesnakes to have high variability in their venom composition.

Rattlesnakes possess various different toxins from 10–20 protein families [7,30,40,41,42]. These families possess several enzymes, such as: L-amino acid oxidases (LAAO) [30,43,44,45,46], phosphodiesterase (PDE) [47,48,49], snake venom metalloproteases (SVMP) [50,51,52], serine proteases (SVSP) [44,53,54,55], phospholipases (PLA_2_) [56,57,58,59].Additionally, rattlesnake venoms also contain nonenzymatic proteins like myotoxin a and its homologs [60,61,62,63], bradykinin-potentiating peptides and bradykinin-inhibitory peptide (BPPs and BIPs) [30,44,64,65], disintegrins (Dis) [3,44,45,55,66,67,68], cysteine-rich secretory proteins (CRiSPs) [2,45,55,62], and C-type lectins (CTL) [30,41,48,69]. It is not uncommon to have variation in venom composition within species [17,23]. This plasticity and variability of venom gives it a unique advantage in biomedical research.

Even though venom enjoys a unique advantage in biomedical research, it is plagued by three main issues: (i) absence of data on venom composition of several species within *Crotalus* genus (rare and/or topographically inaccessible species); (ii) high-cost associated with venom-based studies; (iii) high variability in venom composition rendering venom composition studies for all age classes in all populations of rattlesnakes impractical; and (iv) sparse data on the relationship between various venom components. These factors manifesting together often make it difficult to predict the venom components in any species. A natural solution to this issue is quantifying the relationship between venom components rather than individual units. For example, within *Crotalus polystictus*, the type and potency of proteins expressed vary with age and sex [48,69,70,71]. Many venom components discovered, such as LAAO and PDE, have not been explored for their potential biomedical applications [32,72]. However, based on the associations and ubiquity alone, it is evident that they do play a role during envenomation.

These new-found relationships between various protein components in snake venom may play key roles in developing suitable treatments for prevalent diseases. Such relationships between toxins, often termed synergisms, are joint effects of multiple toxins, which assert greater effects than the sum of individual potencies, thus allowing the individual components to be highly effective with only trace amounts [73,74]. In the current study, we conduct a meta-analysis to understand the relationship between various protein components in *Crotalus* and *Sistrurus’* venoms. We also report the frequency with which various proteins occur in *Crotalus* and *Sistrurus’* venoms. Here we re-classify venom components as major or minor based on their medical relevance, synergies with other toxins, frequency of occurrences.

## 2. Result

Our search consisting of six keywords and 14 databases produced 192 studies on *Crotalus* and *Sistrurus* venom. Out of these 192 studies, 77 studies are not included in the current meta-analysis; 36 out of 77 studies did not meet inclusion criteria, and 41 studies met the exclusion criteria. The remaining 115 full-text articles are included in the analysis. From these 115 articles, only 47 reported a relative abundance of venom components for *Crotalus* and *Sistrurus* species.

### 2.1. Venom Constituents in Crotalus Venom

We identified compositional venom studies, through both transcriptomic and proteomic technologies, for 30 entries, including species and subspecies, within the genus *Crotalus*. 46 protein families are present in *Crotalus* (Table 1). These protein families could be classified based on ubiquity or relationship with other proteins. There is little information regarding the venom composition of nine *Crotalus* species and subspecies (Table 1). 41 studies reported the relative abundance of protein constituents.

#### 2.1.1. Frequency of Protein Components in *Crotalus* Venom

**Figure 1:**
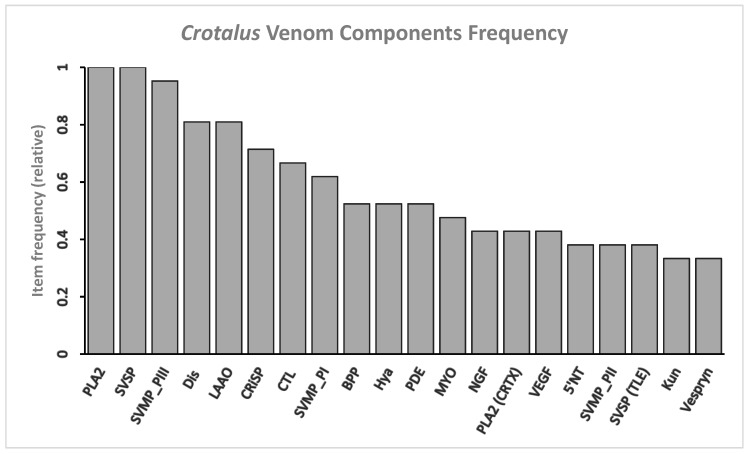
Twenty most common venom components in venom expressed by genus: *Crotalus*. PLA_2_ and SVSP are identified as the most common among *Crotalus* species (relative frequency is 1). Note: 5′-nucleotidase (5′-NT), bradykinin inhibitory peptide (BIP), bradykinin potentiate peptide (BPP), C-type lectins (CTL), natriuretic peptide type C (CNP), crotoxin (CRTX), disintegrin (Dis), guanylyl cyclase (GC), hyaluronidase (Hya), Kunitz-type inhibitor (Kun), L-amino acid oxidase (LAAO), myotoxin (MYO), nerve growth factor (NGF), phosphodiesterase (PDE), phospholipase a_2_ (PLA_2_), phospholipase b (PLB), snake venom metalloprotease (SVMP), snake venom serine protease (SVSP), vascular endothelial growth factor (VEGF).

The ubiquitous protein families in *Crotalus* venom are PLA_2_, SVMP P-III, SVSP, Dis, LAAO, CRiSP, CTL, SVMP P-I, BPP, Hya, and PDE (Figure 1).

#### 2.1.2. Association between Various Venom Components in *Crotalus* Venom Using Presence/Absence Data

Using the frequent item-set approach from data-mining literature [164], we identify a total of 559 relationships between different venom components for *Crotalus* (Table S1) (first three rules did not identify the predictor protein(s) and hence are discarded). See Box 1 for further discussion of terms associated with frequent item-set data-mining.

Box 1 **Frequent item-set data-mining.**

First introduced in 1993 [165], association rule mining has emerged as a popular technique in detecting and extracting key structural information from large-scale transaction data that is often generated in organizations, such as Krogers, Walmart, etc. [166]. These rules help the organizations understand the co-occurrence patterns and frequencies of various transactions, thus helping them become more efficient and profitable. By leveraging the similarity between the co-occurrence of protein components in venom, and transactions done by various shoppers in these supermarkets, we can use the powerful data-mining tools for discovering patterns in venom composition to boost the efficiency of biomedical research.

Let I = { i_1_, i_2_, i_3_,…, i_n_) be a set of n protein components (referred to as “items” in data-mining literature) and D = {t_1_, t_2_, t_3_,…, t_n_} be the set of venom samples. An association rule is defined as an implication of the form X => Y where X, Y ⊆ I and X ∩ Y = ∅. The set of protein components (referred to as item sets) X and Y are called antecedent (left–hand side LHS or predictor) and consequent (right-hand side RHS or predicted) of the rule [167].

The support (supp) (X) of itemset X is defined as the proportion of venom samples in the data set which contain the itemset.

Confidence of a rule is defined as conf (X => Y) = supp (X ∪ Y)/supp (X) [167]. Confidence can be interpreted as an estimate of the probability P (Y|X), the probability of finding RHS of the rule in the venom sample under the condition that these venom samples also contain LHS [167].

Lift of a rule is defined as (X => Y) = supp (X ∪ Y)/(supp(X)supp(Y)). It can be interpreted as the deviation of the support of the whole rule from the support expected under independence given the supports of the LHS and RHS [167]. Greater lift values indicate stronger associations [167].

**Figure 2:**
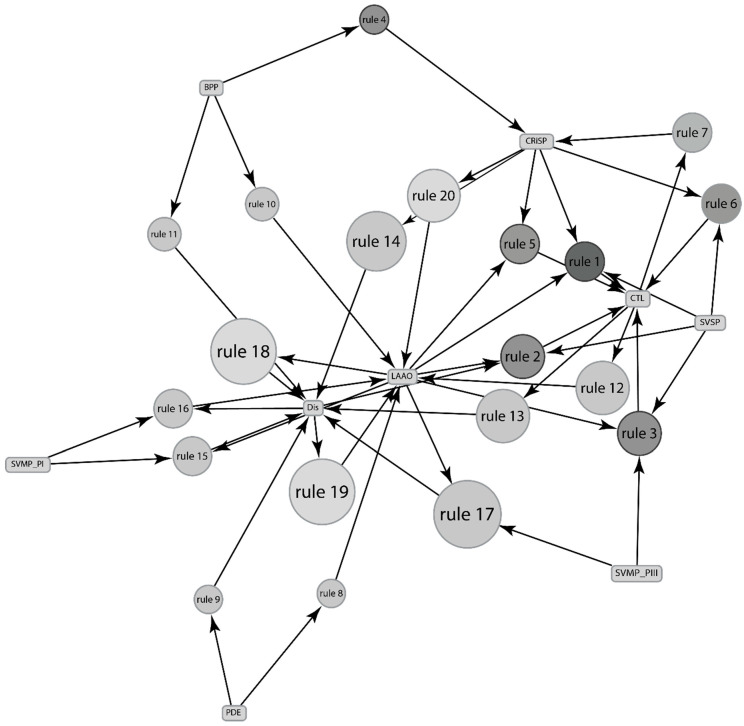
Depictions of association between components of venom expressed by genus *Crotalus.* The rules are depicted by the top twenty minor components-related rules as stated in Table 2. The size and the depth of color of the graph nodes are proportional to the support level and lift ratios of the underlying association rules.

In this study, we highlight the top 20 associations (Figure 2), e.g., Dis is associated with CTL with a confidence of 1 and support of 0.667 (Table 2), implying that Dis and CTL are expressed together 66.7% times in venom of all species of *Crotalus*, and if CTL is expressed in venom, then Dis is expressed 100% times.

*Crotalus’* venom components are well studied to generate more than 500 associations, but only the top twenty relevant rules with at least 1 minor component are depicted in Table 2. If protein (predictor) is present in venom, then chances of the protein (predicted) to be expressed in the venom are given by combining “confidence” and “lift”. Dis has the highest number of associations as a predicted component, which is 7: PDE, BPP, CRL, CRiSP, LAAO, SVMP P-I and LAAO, SVMP P-III and LAAO. Followed by LAAO with six associations: PDE, BPP, CTL, Dis and SVMP P-I, Dis, and CRiSP. On the other hand, CTL is associated with five groups, and CRiSP is represented by two associations. However, 5 associations of CTL have higher lift and confidence than LAAO’s and Dis’, indicating better associations.

#### 2.1.3. Association between Various Venom Components in *Crotalus* Venom Using the Relative Abundance of Protein Components

**Figure 3:**
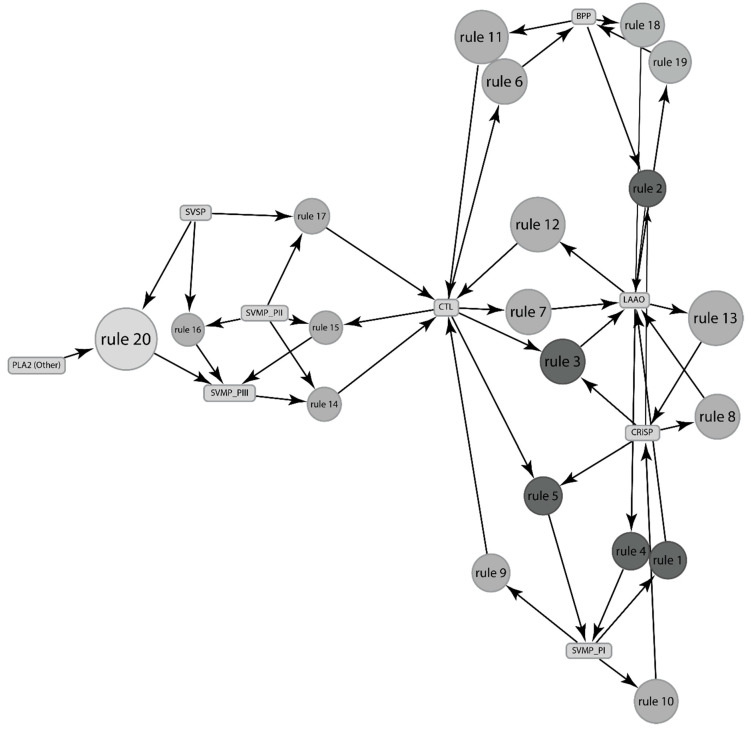
Using maximum values of relative abundances of the venom components, the association between components of venom expressed by genus *Crotalus.* The rules are depicted by the top 20 rules, as shown in Table 3. The size and the depth of color of the graph nodes are proportional to the support level and lift ratios of the underlying association rules.

A key challenge in inferring association between different species is the lack of data on relative abundance for venom components, e.g., for the *Crotalus* species, relative abundance is reported only for 14 out of the 30 species. Within these 14 species, relative abundance is reported for only 56.7% of the venom components. Using the limited data on relative abundances, we can identify a total of 47 association rules, also referred to as relationships between different venom components for *Crotalus* (Table S2). Herein we report only the top twenty relevant rules (Table 3, Figure 3).

Despite limited data, many relationships reported through presence/absence data are also reported through relative abundance data, such as CTL and LAAO, CTL and SVMP-PIII, CTL and SVSP, BPP and LAAO, and CRiSP and LAAO. Through relative abundance data, we identified several new relationships, such as PLA_2_ and SVMP_PIII, SVMP_PII and CTL, etc. (Figure 3).

#### 2.1.4. Hierarchical Clustering of Venom Components to Identify any Similarity or Dissimilarity with Phylogenetic Relationships

**Figure 4:**
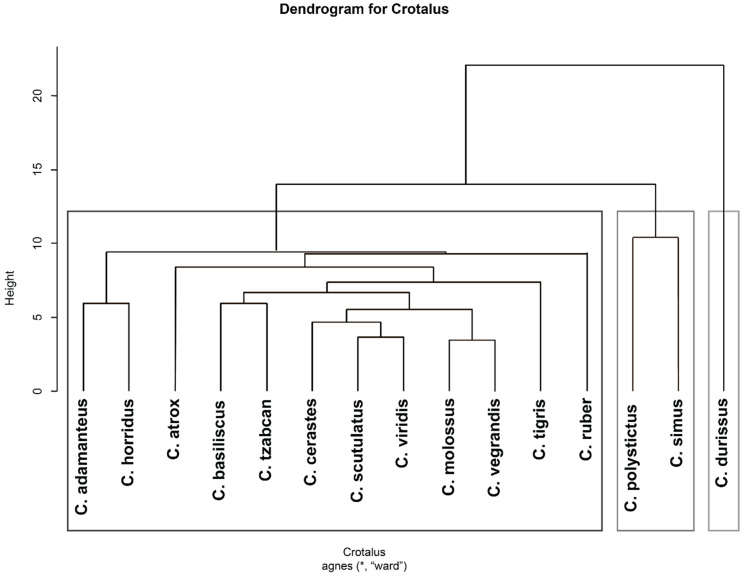
Hierarchical clustering of *Crotalus* species using maximum relative abundance values of known venom components. * represents the dataset used for genetating this dendogram.

We used hierarchical clustering analysis of venom components with known relative abundances to cluster different *Crotalus* species according to the similarity in venom components (Figure 4). We found the clustering similar when we used maximum reported values of relative abundances for each venom component within a species to the average relative abundances of venom components within a species. One would expect similar venom composition in closely related species due to recent common ancestor, but such similarity was not observed. We conjecture that the reason different species have similar compositions is due to functional similarities. The venom composition of *Crotalus durissus* is different from the rest of the 14 species. *Crotalus polystictus* and *Crotalus simus* have similar venom composition, while *Crotalus atrox*, *Crotalus bassilliscus*, *Crotalus tzabcan*, *Crotalus cerastes*, *Crotalus scutulatus*, *Crotalus viridis*, *Crotalus molossus*, *Crotalus vergandis*, *Crotalus tigris*, *Crotalus ruber*, *Crotalus horridus* have similar venom composition (Figure 4).

### 2.2. Venom Constituents in Sistrurus Venom

We identified compositional venom studies, through both transcriptomic and proteomic technologies, for 34 entries, including species and subspecies, within the genus *Sistrurus*. Few studies have focused on the *Sistrurus* subspecies’ venom. 19 protein families are present in *Sistrurus* (Table 4). These protein families could be classified based on ubiquity or relationship with other proteins.

#### 2.2.1. Frequency of Protein Components in *Sistrurus* Venom

**Figure 5:**
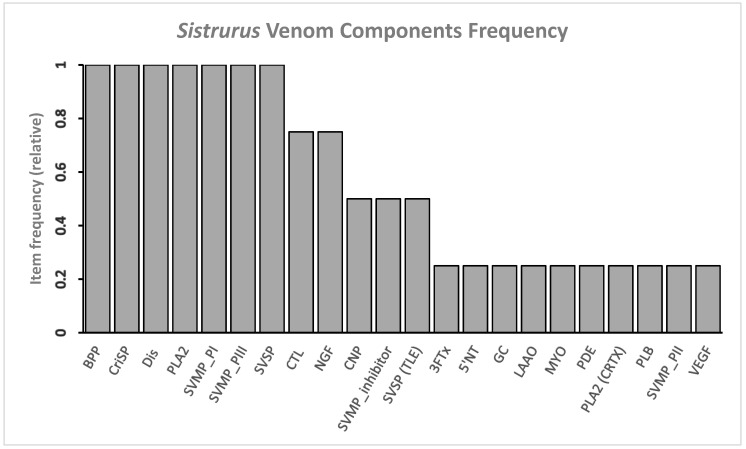
Twenty most common venom components in venom expressed by genus: *Sistrurus*. BPP, CRiSP, Dis, SVMP P-I/III are the most common toxins (relative frequencies are 1). Note: three-finger toxin (3FTx), 5′-nucleotidase (5′-NT), bradykinin potentiate peptide (BPP), C-type lectins (CTL), natriuretic peptide type C (CNP), cysteine-rich secretory protein (CRiSP), crotoxin (CRTX), disintegrin (Dis), guanylyl cyclase (GC), L-amino acid oxidase (LAAO), myotoxin (MYO), nerve growth factor (NGF), phosphodiesterase (PDE), phospholipase a_2_ (PLA_2_), phospholipase b (PLB), snake venom metalloprotease (SVMP), snake venom serine protease (SVSP), vascular endothelial growth factor (VEGF).

The dominant protein families based on ubiquity in *Sistrurus* are BPP, CRiSP, Dis, SVMP, CTL, NGF, PLA_2_, and SVSP (Figure 4). The main difference between *Crotalus* and *Sistrurus* proteins is due to the absence of 27 venom components in *Sistrurus* (Figure 1 and Figure 5). Some of the absent venom components from *Sistrurus*’ proteomic and transcriptomic are: alkaline phosphomonoesterase (APase), acetylcholinesterase (achase), aminopeptidase, angiogenin, natriuretic peptide (ANP and BNP), ATPase, bradykinin inhibitory peptide (BIP), platelet-derived growth factor (PDGF), carboxypeptidase, cysteine protease (CysProt) and CysProt inhibitor, dipeptidase, dipeptidyl peptidase, EF-hand protein, epidermal growth factor (EGF), exendin4-like protein, endonuclease (DNAse and RNAse), fibroblast growth factor (FGF), ficolin/veficolin, glutathione peroxidase, hyaluronidase (Hya), Kazal-type inhibitor (Kazal), Kunitz-type inhibitor (Kun), lipase, ohanin (OHA), platelet-derived growth factor (PDGF), vespryn, phospholipase d (PLD), and waparin (WAP).

#### 2.2.2. Association between Various Venom Components in *Sistrurus* Venom Using Presence/Absence Data

**Figure 6:**
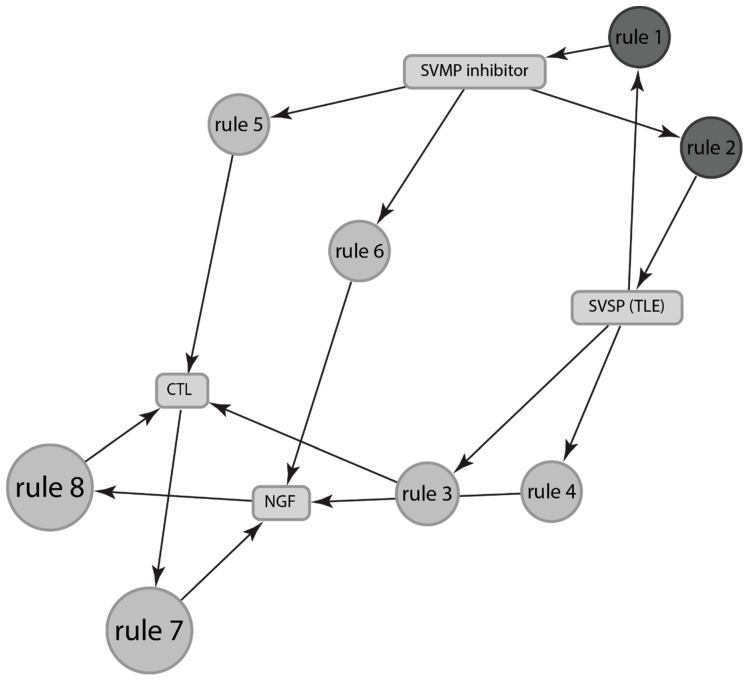
Depictions of association between components of venom expressed by genus *Sistrurus.* The rules are depicted by the top eight rules as stated in Table 5. The size and the depth of color of the graph nodes are proportional to the support level and lift ratios of the underlying association rules.

Using the frequent item-set data mining approach from data mining literature [164], we can identify eight relationships between different venom components for *Sistrurus* (Figure 6), e.g., NGF is associated with CTL with confidence = 1, support = 0.75 (Table 5), implying that NGF and CTL are expressed together 75% times in venom of all species of *Sistrurus*, and if NGF is expressed in venom, then 100% times CTL is also expressed.

In contrast to *Crotalus’* venom components, studies on *Sistrurus’* venom component are lacking, and thus, only a small pool of studies are used to generate only eight associations, as depicted in Table 5. CTL and NGF each have three associations with different venom components. CTL is associated with NGF, SVMP inhibitor, and SVSP; NGF is associated with CTL, SVMP inhibitor, and SVSP. They are followed by SVMP inhibitor and SVSP with one association each: SVMP inhibitor is associated with SVSP and vice versa. However, SVMP inhibitor and SVSP’s associations have higher lift and confidence than CTL’s and NGF’s, indicating better associations.

#### 2.2.3. Association between Various Venom Components in *Sistrurus* Venom Using the Relative Abundance of Protein Components

Similar to *Crotalus*, a key challenge in inferring association between different species is the lack of data on relative abundance for venom components. There are only six studies that reported relative abundances for all species and subspecies within the genus *Sistrurus*. Relative abundance is reported for 21 out of 25 venom components. We identified a total of 13 associations (Table 6). Nine associations are discarded as they did not have any predictor component or are duplicates.

**Figure 7:**
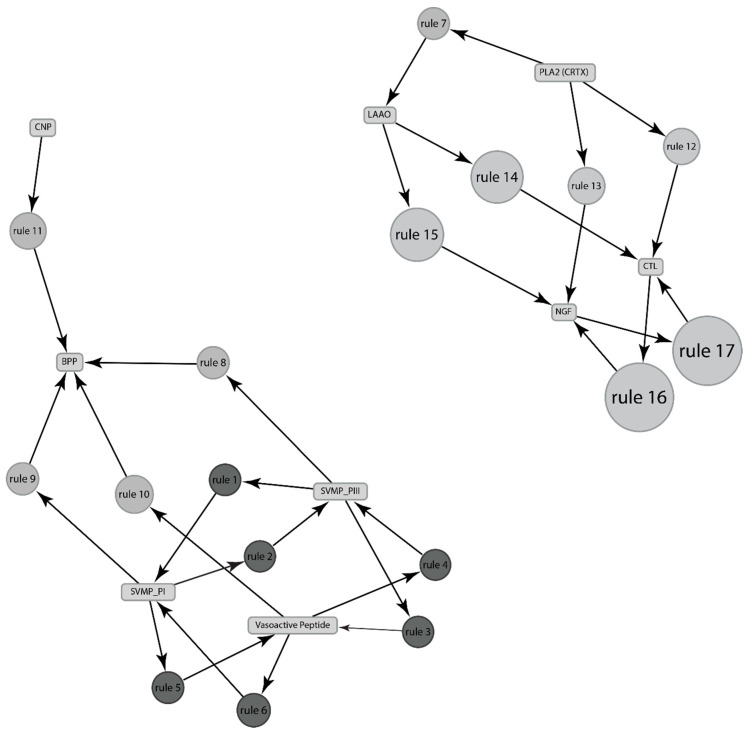
Using maximum values of relative abundances of the venom components, the association between components of venom expressed by genus *Sistrurus.* The size and the depth of color of the graph nodes are proportional to the support level and lift ratios of the underlying association rules. The rules are depicted by the top 13 rules as stated in Table 6.

Unlike presence/absence data, we found several new associations, such as PLA_2_ associated with LAAO, CTL, and NGF; SVMP_PIII associated with SVMP_PI, Vasoactive peptide, and BPP, etc. (Table 6, Figure 7).

#### 2.2.4. Hierarchical Clustering of Venom Components to Identify Similarities or Dissimilarities in Phylogenetic Relationships

**Figure 8:**
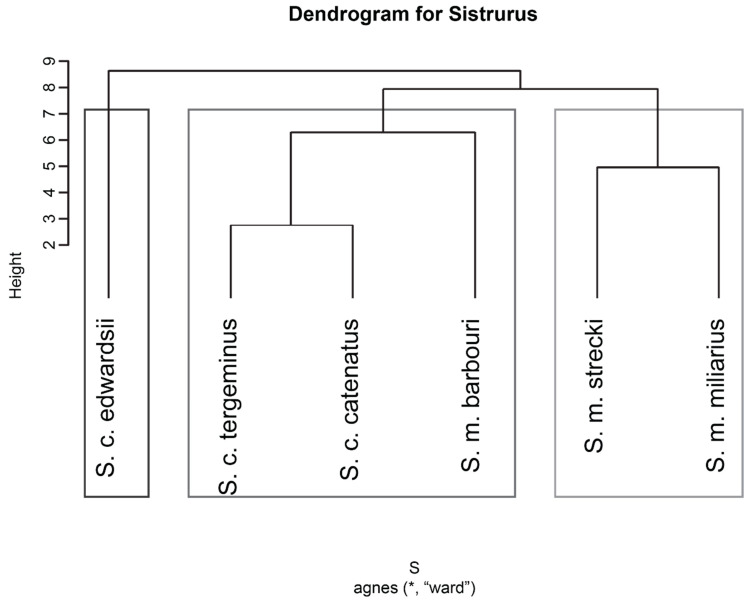
Hierarchical clustering of *Sistrurus* species using maximum relative abundance values of known venom components. * represents the dataset used for genetating this dendogram.

Next, we use hierarchical clustering analysis of venom components with known relative abundances to cluster different *Sistrurus* species according to the similarity in venom components (Figure 8). One would expect similar venom composition in closely related species due to recent common ancestor, but such similarity is not observed. We conjecture that the reason different species have similar compositions is due to functional similarities. We found that the venom compositions of *Sistrurus miliarius miliarius* and *Sistrurus miliarius strecki* are similar. However, *Sistrurus miliarius barbouri*, phylogenetically similar to *Sistrurus miliarius miliarius* and *Sistrurus miliarius strecki*, does not have similar venom composition to these two subspecies of *Sistrurus miliarius*. Instead, its venom composition is similar to that of *Sistrurus catenatus tergeminus* and *Sistrurus catenatus catenatus. Sistrurus catenatus edwardsii* have a different venom composition than the other two subspecies (Figure 8).

## 3. Discussion

A total of 46 families of proteins are identified in the venom of 34 species and subspecies of rattlesnakes. Most studies focus on *Crotalus*, and a subset of studies focus on *Sistrurus.* Through our analysis, using the presence/absence of venom components, we can discover a total of 562 association rules for *Crotalus* and 25 association rules for *Sistrurus* venom components. In this study, we present the 20 most relevant rules for *Crotalus* and eight rules for *Sistrurus* venom components, respectively (Table 2 and Table 5). Using the known relative abundances of venom components, we discovered 47 rules for *Crotalus* and 13 rules for *Sistrurus* venom components (Table 3 and Table 6).

Using presence/absence data in developing venom component association only gives limited insight as venom becomes functionally different with changes in relative abundances of its components. However, we have been limited by existing information on the relative abundances of venom components. Within *Crotalus*, relative abundances have been reported for 46% of the species, and within these species, relative abundances have been reported for only 56% of venom components. Within *Sistrurus*, for all species and subspecies, relative abundances have been reported for 84% of venom components. Reporting relative abundances of different venom components would play a critical role in developing more insightful associations between different venom components.

There is an emphasis on investigating venom components stand-alone units with a lack of investigations of their relationships with each other and the subsequent effects of co-administering different components. On the other hand, understanding the relationship between venom components could open a new avenue for biomedical research and unlock protein combinations that yield enhanced bioactivity in pharmaceutical drugs. Additionally, studying components as stand-alone may have produced a negative effect in which many components have received skewed attention in biomedical research. For example, protein families are often classified as major or minor based on importance and ubiquity [13,128]. Thus, causing the dominant protein families, such as proteases, neurotoxins, and phospholipases, to be more researched than other protein families, such as growth factors. However, it is by combining ubiquity, bioactivity, and relationship between the protein families that we can classify the venom components as major or minor.

In rattlesnakes, MYO, PLA_2_, SVMP, and SVSP are classified as major components based on medical importance and ubiquity [13,128], which is also confirmed by our analysis (Figure 1). However, with a new approach of using both ubiquity and number of associations for each protein, we find that Dis, LAAO, CTL are all more ubiquitous and have more associations with other proteins in *Crotalus* species (Table 2). Similarly, in *Sistrurus* species, the SVMP inhibitor and NGF (Figure 4) have the most associations than MYO, which has only one association (Table 3).

These associations play a critical role in the synergy between venom components [73]. This synergism causes the joint effects of multiple toxins to assert greater effects than the sum of individual potencies [73], making trace amount of snake venom to be highly efficient and effective [73,74]. Such combinations of venom proteins often cause various symptoms of bleedings, tissue degradation, necrosis, and further complications in prey and bite victims [69,177] and improve the lethality of whole crude venom in contrast to individual components [73,178].

Through mostly studies of predominant toxins, different general mechanisms for toxin synergisms have been proposed [73,179]:

(1) Two or more toxins interact with different targets on related biological pathways, resulting in synergistically increased toxicity;
(2) Two or more toxins recognize and interact with the same target synergistically and produce the same effect, and is often called amplification;
(3) One toxin (subunit) acts as a chaperone to potentiate another one. The chaperone may expose the active/functional site of the second toxin (subunit), or expose target sites, or increase affinity to target or modify the active surface of the other toxin (subunit). Such complexes usually dissociate after asserting their toxicity.

Synergisms are mostly reported for major toxins in rattlesnake venoms [73]. A notable example of synergism through complex formation (mechanism 3) is crotoxin, a lethal neurotoxin from *C. durissus terrificus*, by two subunits: an acidic subunit component A (CA or crotapotin) and a basic subunit component B (CB) [109,115,116,180,181]. CB is identified as a basic PLA_2_ with phospholipase activities and low toxicity, while the CA component is said to be a small acidic, nonenzymatic, nontoxic subunit [73,181]. However, once combined non-covalently, CA improves the potency of CB by enabling CB to reach the specific crotoxin receptors at the neuromuscular junction as well as inhibits other CB functions, such as catalytic and anticoagulant activities [115,181]. Thus, the resulting crotoxin complex is highly active, compared to individual components, showing the synergy between two subunits blocking acetylcholine release [180,181]. Similarly, in *C. scutulatus scutulatus*, the Mojave toxin is another PLA_2_ complex: one acidic and nonlethal subunit acts as a chaperon for the other basic subunit to improves lethality [41,148,162]. Other examples of synergistic complexes have been found and reported in many species of Viperidae and Crotalidae [73]. Such interactions show the strong synergistic activities in rattlesnake venoms that have been studied intensively through previous endeavors.

A prevalent example between major components is SVMP P-III and an acidic PLA_2_ in *Bothrops alternatus* called baltergin and Ba SpII RP4 PLA_2_, respectively [182,183]. The more abundant PLA_2_ has no myotoxic activities, while the less abundant baltergin possesses high edematogenic and myotoxic activities [182], while PLA_2_ has no myotoxicity, although it is the most abundant PLA_2_ in this species [183]. When acting simultaneously, both can cause complete detachment of C_2_C_12_ myoblast cells, while none can achieve 50% of detachment on their own [184]. The analogous synergism has also been recorded in endothelial cells, SVMP’s natural target [73,185]. The mechanism of synergism for such interaction is proposed through interactions with endothelial cells’ membranes, free of catalysis rather than enzymatic activities of PLA_2_ [185]. Since PLA_2_ does not target extracellular matrix proteins like SVMP [182], indicating that the second general mechanism of toxin synergism is followed. Both enzymes are present in many rattlesnakes’ venoms (Table 1 and Table 3), and their association is also reported through our analysis (Table S1). There are reports indicating the synergism between crotoxin and crotamine, a member of MYO toxins in *Crotalus* venoms, which facilitates the internalization of the CB subunit and increases neuronal toxicity [73,186]. Unfortunately, these interactions are not found in the analysis (Table 2), although they are present in *Crotalus* venoms (Table 1), which could be due to the sparse reports on *Crotalus’* venoms with many species are still under-investigated as stated previously.

Even fewer studies focus on the synergism between major and minor components: SVSP, a major toxin, and BPPs, a minor toxin [73], indicating a biased approach in studying venom toxins produced by the current major/minor toxin classification convention. BPPs, which are micromolecular hypotensive peptides in snake venoms, can inhibit angiotensin-converting enzymes and induce hypotensive action of bradykinin, accompanied by hyperpermeability of blood vessels [65,107,187,188]. Thus, BPPs are targeted for many pharmaceutical developments to treat hypertension and heart failure [189,190]. On the other hand, many SVSPs show activities that are similar to kallikrein, a serine proteinase, with the specific and limited proteolytic functions that release bradykinin [73,143,191]. Previous works indicated that BPPs could act synergistically with kallikrein-like SVSPs, which release bradykinin more effectively than endogenous kallikrein to produce potent hypotension and vascular shock in prey [73,95,143,192,193,194] (mechanism 1). Similarly, SVSP-BPP interaction results in a stronger physiological effect than from individual components. However, studies dedicated to understanding the mechanism and effects of such interactions are limited. The absence of such studies highlights the bias in current classification systems of major and minor venom components. Likewise, there are many components (e.g., LAAO) with substantial associations with other toxins, like CTL or NGF, that have not been investigated for their potential synergisms. Therefore, there is a need to develop a deeper understanding of minor components in the venom of rattlesnakes to discover more associations, such as that of SVSP and BPP.

Another way to explain the associations of these toxins is through the evolution of toxins. One relationship that has been explored in previous studies is between SVMP P-III and Dis. Dis is a small, nonenzymatic protein that can bind to extracellular receptors (integrins) with many motifs and sizes, two of which are RGD and MVD motifs [9,68,144,195,196]. While SVMP-PIII is a subclass of SVMP with a Dis-like domain [197,198], Dis, especially the RGD/MVD motifs, is suggested to be produced from the rapid evolution of the genes coding of SVMP-PIII [195,196,199]. The RGD/MVD motifs of Dis are presented in many *Crotalus* species [195] along with SVMP-PIII, represent as rule 17 (Table 2) can be explained through this evolution model, although the co-association with LAAO is still largely unknown.

Some associations may not need to be derived through their toxicity but could be explained through the proteins’ housekeeping functions. The existence of SVMP inhibitor is thought to be a housekeeping molecule, despite its potential therapeutic activities, which helps neutralize the potent SVMP in the venom glands as a self-defense mechanism [200]. Yet, not many studies have been invested in this family, along with the lack of occurrence in many rattlesnake venoms, where high amounts of SVMP exist (Table 1), which indicates a knowledge gap that requires further investigations. Likewise, NGF is known for its ability to inhibit SVMP proteolysis in Viperidae [201,202]. However, growing evidence has suggested the plausibility of other mechanisms in which NGF can act as cytotoxic proapoptotic factors in tissues that do not have TrkA receptors [201,203,204]; or as ancillary functions, like Hya, to help with efficient absorption of venom component through the release of granules molecules (histamine, serotonin, etc.) [179,201,205]. Such large release can also have impactful consequences (anaphylaxis, bronchoconstriction, vasodilation, etc.) [201,206]. However, not many *Crotalus* species have NGF, as observed previously in Table 1, indicating yet another gap of knowledge in *Crotalus* venomics. Using SVMP as a common targeting model to explain the association of NGF and SVMP inhibitor (rule 6, Table 5) is promising, but due to the insufficient amount of information provided, such explanation warrants further attempts in co-administration testing to confirm.

Developing phylogenetic relationships between different species using venom composition would further explain the various associations between different venom components as two species can have similar venom composition due to recent common ancestor or functional reasons. The existing venom composition data is greatly insufficient for developing the phylogenetic tree (Figure 4). However, the hierarchical clusters developed using known relative abundances show a different relationship than the observed phylogenetic relationships [207]. Even though *C. durissus* is similar to *C. basiliscus*, the venom composition of the latter is much more similar to that of *C. molossus*. The venom composition of *C. durissus* is unique compared to other species (Figure 4). Similar patterns can be observed with *Sistrurus* species (Figure 8). The possible explanations for this may be either functional similarity or insufficient data. However, one would expect the cluster structure to change with changes in venom composition due to age, sex, diet [17,18,24,25], or topographical features [26,27]. We did not observe any difference between clusters built using the maximum relative abundance value or the average values. However, this result should be taken with caution as only a few studies reported multiple relative abundance values for some of the venom components. Thus, to have a deeper insight into the venom associations and venom component relationships, relative abundances must be quantified and reported.

## 4. Conclusions

In this paper, we have elicited the associations between different venom components. Thus, expediting future research on the synergy between various venom components. We also establish the need to report relative abundances for different venom components to increase the accuracy of the predicted associations and the understanding of venom evolution.

The results of this study suggest a myriad of associations, many of which are yet to be discovered, but they do provide promising potential synergistic effects that are worth further investigation. For example, using rules 2 and 13 in *Crotalus* venoms (Table 2), CTL, a protein/glycoprotein that specifically binds to carbohydrate moieties and glycoconjugate, can target and interact with platelet receptors and blood coagulation factors [208], which are also targets for Dis [209], indicate their potential synergisms with antiplatelet toxins and assert the hypotensive results along with many other toxin groups like SVSP [73,95,143,192,193,194]. Thus, highlighting the importance of characterizing toxin components and their associations [69]. With an increased amount of characterization studies, novel families may also be correctly added into the venom profiles, such as three-finger toxins (3FTx), which often are present in elapids and a few occasions in rattlesnakes genome and transcriptome [14,15,16,169,171]. However, attention should be paid to developing venom profiles for understudied genera (*Sistrurus*) or species. Additionally, this work also addresses the problem of conventional classification of venom toxins as major or minor based on importance and ubiquity, which are often MYO, PLA_2_, SVMP, and SVSP [13,128], as the cause of much more attention on these dominant toxin families and overlooking other protein families, such as Growth factors. Therefore, we highlight the importance of studying venom components not only as individual components but also in understanding the relationship between them. We propose using the combination of toxin’s characteristics, such as its ubiquity, bioactivity, and associations with other toxin families, to classify the venom components as major or minor.

## 5. Materials and Methods

We collected articles and abstracts on venom for each *Crotalus* and *Sistrurus* species through the following: databases (PubMed, ScienceDirect, Scopus, Google Scholar, Web of Science), journal’s databases (*BMC Genomics*, *Journal of Proteome Research*, *Journal of Proteomics*, *Toxicon*, *Toxicology*, *Toxins*), publisher databases (Wiley Online Library, MDPI, Elsevier). We used “venom” OR “proteomic” OR “venomic” OR “transcriptome” OR “proteome” AND “name of the species” as keywords for conducting our search. We also examined references in studies produced from the search results for any additional information. Collected records were the earliest obtainable records to those that are published in January of 2020.

From collected records, any article that did not contain information regarding venom composition and components of any *Crotalus* and *Sistrurus* species was not used in the current analysis. Otherwise, the articles’ full-text version would be further assessed with the following inclusion and exclusion criteria.

For the article to be included in the current analysis, it had to fulfill one of the following inclusion criteria: (1) report proteome or transcriptome profile of the venom of any corresponding species; (2) report at least 1 toxin family/component, which is not artificially synthesized based on another similar toxin component; (3) be a comparative study reporting transcriptome/proteome profile for *Crotalus*, *Sistrurus* species/subspecies; (4) studies that report variability in venom components for any *Crotalus*, *Sistrurus* species/subspecies.

The following exclusion criteria were used to exclude any study from the current analysis: (1) reviews that focus on toxin families and/or articles focuses on the genomic evolution of toxin families; (2) articles with no transcriptome/proteome profiles; (3) articles with no data on toxin family isolated from venom; (4) articles that focus on new artificially synthesized molecules, based on similar toxin component or recombinant protein/peptides in venom; (5) articles reporting methods to inactivate toxin family from rattlesnakes; (6) case study on rattlesnakes’ bites; (7) studies describing methods to detect toxin families/components. From the studies that fulfilled our inclusion criteria and did not meet any exclusion criteria, we collected and compiled all venom constituents that are reported for each species in the genus *Crotalus* and *Sistrurus* in Table 1 and Table 4 respectively. The compiled data were cross-checked by authors for correctness and confirmations.

Using the data from Table 1 and Table 4, we performed two separate frequent item-set data mining analyses for *Crotalus* and *Sistrurus* venoms. We conducted frequent item-set data mining using presence-absence data and a separate analysis using relative abundance values. In the analysis using relative abundance values, when more than one value for relative abundance was reported for a particular protein, we used the maximum value of relative abundances reported. There was no major difference in the results when we used maximum reported values *versus* the average of all reported values for a particular protein. For all values that are reported as below the limit of detection, we used the limit of detection as the value for that particular component [210]. Frequent item-set data mining helps identify the association rules associated with the expression of different proteins in venom. Studies on *Sistrurus* venom components are sparse, thus, can introduce a bias towards data-mining analysis. The rules specify the confidence, lift, and support for specific proteins to occur together in venom. Support is defined as absolute frequency, i.e., a support of 25% means that venom components x, y, and z occur together in 25% of all venoms. Confidence is correlative frequency., i.e., a confidence of 60% means that if x and y occur, then 60% of times z will also occur. Lift signifies the likelihood of the y occurring when x occurs while taking into account the number of times venom component y occurs in different species. An association rule is valid only if the lift is greater than 1. The higher the value of the lift, the higher is the validity of the rule. Since many studies associated with rattlesnake venom concentrated on highly abundant species or species containing more “major components”, this affects the performance of the statistical models due to the presence of null values. For the analysis using only the presence–absence data for toxin families from individual studies, the chances of bias from individual studies affecting our results were low. With the increase in venom composition and variation data, the associations produced by frequent item-set data-mining analysis will be more informative. Using the relative abundance data of venom components from Table 1 and Table 4, we performed hierarchical clustering for both *Crotalus* and *Sistrurus* species. For species with multiple values reported for the same venom component, we used the maximum of all reported values in our analysis. All analysis was performed using the software R (R Core Team, Vienna, Austria, 2019).

## Figures and Tables

**Table 1:** Venom components within the *Crotalus* genus.

Species	Venom Components	Reference
*C. adamanteus*	5′NT, BPP, carboxypeptidase (E-Like), CNP, CRiSP, CTL, dipeptidase, Dis, EF-hand protein, EGF, GC, Hya, Kun, LAAO, MYO, NGF, PDE, PLA_2_, PLB, SVMP-P I/II/III, SVSP, VEGF, vespryn	[75,76,77,78,79,80,81,82,83,84,85,86,87]
*C. aquilus*	Hya, PLA_2_, SVMP P-III, SVSP (TLE)	[59,69]
*C. atrox*	BIPs, BPPs, CNP, CRiSP, Dis, Hya, LAAO, CTL, PLA_2_, SVMP P-I/III, SVSP, VEGF	[6,44,80,88,89,90,91,92,93,94,95]
*C. basiliscus*	BPP, CRISP, CTL, Dis, LAAO, PLA_2_ (CRTX, non-CRTX), SVMP P-I/II/III, SVMP inhibitor, SVSP	[96,97,98,99]
*C. catalinesis*	SVSP, SVMP P-III, PLA_2_	[40]
*C. cerastes*	3FTx, 5′NT, BPP, CRiSP, CTL, Dis, ficolin, Hya, Kun, LAAO, MYO, NGF, PDE, PLA_2_, SVMP P-II/III, SVSP, VEGF, vespryn, WAP	[98,100,101,102]
*C. durissus*	3FTx, achase, aminopeptidase, angiogenin, BPP, carboxypeptidase, CNP, CRiSP, CTL, CysProt inhibitor, CysProt, dipeptidyl peptidase, Dis, FGF, fraction 5, Hya, Kazal, Kun, LAAO, lipase, MYO, NGF, PDGF, PLA_2_ (non-CRTX, CRTX), PLB, PLD, Serpin-like, SVMP inhibitor, SVMP P-III, SVSP, VEGF, vespryn, WAP	[45,64,103,104,105,106,107,108,109,110,111,112,113,114,115,116,117,118]
*C. enyo*	SVSP, SVMP P-I/III, PLA_2_	[40]
*C. horridus*	5′-NT, BPP, CNP, CRiSP, Dis, EGF-like, GC, Hya, Kun, LAAO, MYO, neurotrophic factor, NGF, PDE, PLA_2_, SVMP P-I/III, SVSP, VEGF, vespryn	[29,119,120]
*C. lepidus*	5′NT, CRiSP, CTL, Dis, LAAO, PDE, PLA_2_, SVMP-P-I/III, SVSP (TLE, kallikrein)	[49,121,122,123]
*C. mitchelli*	LAAO, SVSP, PLA_2_ (CRTX/MTX)	[40,124,125]
*C. molossus*	Dis, LAAO, MYO, PLA_2_, SVMP P-I/III, SVSP (TLE)	[58,98,126,127,128,129,130]
*C. oreganus*	ANP/BNP, BPP, CNP, CRiSP, CTL, Dis, Hya, Kun, LAAO, MYO, NGF, PLA2 (D49), PLA_2_, SVMP P-II/III, SVSP, VEGF, vespryn	[51,56,57,131,132,133,134,135,136,137]
*C. polystictus*	BIPs, CRiSPs, CTL, Dis, GC, Hya, LAAO, NGF, PDE, PLA_2_, PLB, SVMP P-I/II/III, SVSP (kallikrein, TLE), vespryn	[48,69]
*C. ruber*	CTL, Dis, LAAO, PDE, PLA2, SVMP P-I/III, SVSP (kallikrein)	[40,47,138,139,140,141,142,143]
*C. scutulatus*	5′-NT, APase, BPPs, CRiSP, CTL, Dis, Hya, Kun, LAAO, MYO, NGF, PDE, PLA_2_ (MTX, non-CRTX), SVMP P-I/II/III, SVSP, VEGF, vespryn	[41,52,63,144,145,146,147,148]
*C. simus*	3FTX, 5′-NT, BIPs, BPPs, CRiSP, CTL, Dis, GC, Hya, Kaz, Kun, LAAO, MYO, NGF, OHA, PDE, PLA_2_ (CRTX, non-CRTX), PLB, SVMP P-I/III, SVSP, VEGF, WAP	[7,54,68,149,150,151]
*C. tigris*	CRiSP, Dis, PLA_2_ (MTX), SVMP P-III, SVSP, VEGF	[55,152,153,154]
*C. vegrandis*	5′-NT, ATPase, BIP, BPP, carboxypeptidase, CNP, CRiSP, CTL, Dis, endonuclease (DNAse, RNAse), exendin4-like protein, glutathione peroxidase, Hya, LAAO, MYO, NGF, PDE, PLA_2_ (CRTX), PLB, SVMP P-II/III, SVSP	[77,155,156,157,158,159]
*C. viridis*	5′-NT, APase, BPP, CRiSP, CTL, Dis, GC, LAAO, MYO, OHA, PDE, PLA_2_ (CRTX, non-CRTX), PLB, SVMP inhibitor, SVMP P-I/II/III, SVSP (TLE, kallikrein)	[42,60,61,62,160,161,162,163]
*C. willardi*	CRiSP, CTL, Dis, LAAO, PDE, PLA2, SVMP P-I/III, SVSP (TLE, kallikrein)	[49,153]
*C. tortugenesis*	N/A	
*C. stejnegeri*	N/A	
*C. tancitarensis*	N/A	
*C. lannomi*	N/A	
*C. pusillus*	N/A	
*C. transversus*	N/A	
*C. triseriatus*	N/A	
*C. unicolor*	N/A	
*C. intermedius*	N/A	

Note: three-finger toxin (3FTx), 5′-nucleotidase (5′-NT), acetylcholinesterase (achase), natriuretic peptide type A (ANP), adenosine triphosphatase (ATPase), bradykinin inhibitory peptide (BIP), natriuretic peptide type B (BNP), bradykinin potentiate peptide (BPP), C-type lectins (CTL), natriuretic peptide type C (CNP), cysteine protease (CysProt), cysteine-rich secretory protein (CRiSP), crotoxin (CRTX), disintegrin (Dis), epidermal growth factor (EGF), fibroblast growth factor (FGF), guanylyl cyclase (GC), hyaluronidase (Hya), kazal-type inhibitor (Kazal), Kunitz-type inhibitor (Kun), L-amino acid oxidase (LAAO), Mojave toxin (MTX), myotoxin (MYO), nerve growth factor (NGF), ohanin (OHA), phosphodiesterase (PDE), platelet-derived growth factor (PDGF), phospholipase A_2_ (PLA_2_), phospholipase B (PLB), phospholipase D (PLD), snake venom metalloprotease (SVMP), snake venom serine protease (SVSP), thrombin-like enzyme (TLE), vascular endothelial growth factor (VEGF), waparin (WAP).

**Table 2:** Depictions of association rules between proteins expressed in *Crotalus* venom. Lift signifies the correlation between different venom components. Confidence shows the percentage in which the predicted venom component occurs with the predictor venom component. Support is the number of transactions in which the desired venom component occurs.

Rule No.	Protein (Predictor)	Protein (Predicted)	Support	Confidence	Lift
1	CRiSP, LAAO, SVSP	CTL	0.61	1	1.5
2	Dis, LAAO, SVSP	CTL	0.67	0.93	1.4
3	LAAO, SVMP P-III, SVSP	CTL	0.67	0.93	1.4
4	BPP	CRiSP	0.52	1	1.4
5	CRiSP, LAAO	CTL	0.61	0.92	1.39
6	CRiSP, SVSP	CTL	0.61	0.92	1.39
7	CTL	CRiSP	0.61	0.92	1.3
8	PDE	LAAO	0.52	1	1.23
9	PDE	Dis	0.52	1	1.23
10	BPP	LAAO	0.52	1	1.23
11	BPP	Dis	0.52	1	1.23
12	CTL	LAAO	0.67	1	1.23
13	CTL	Dis	0.66	1	1.23
14	CRiSP	Dis	0.71	1	1.23
15	LAAO, SVMP P-I	Dis	0.57	1	1.23
16	Dis, SVMP P-I	LAAO	0.57	1	1.23
17	LAAO, SVMP P-III	Dis	0.76	1	1.23
18	LAAO	Dis	0.76	0.94	1.16
19	Dis	LAAO	0.76	0.94	1.16
20	CRiSP	LAAO	0.67	0.93	1.15

Note: 5′-nucleotidase (5′-NT), bradykinin potentiate peptide (BPP), C-type lectins (CTL), cysteine-rich secretory protein (CRiSP), disintegrin (Dis), L-amino acid oxidase (LAAO), nerve growth factor (NGF), phosphodiesterase (PDE), phospholipase a_2_ (PLA_2_), snake venom metalloprotease (SVMP), and snake venom serine protease (SVSP).

**Table 3:** Using maximum values of relative abundances of venom components, the association rules between proteins expressed in *Crotalus* venom. Lift signifies the correlation between different venom components. Confidence shows the percentage in which the predicted venom component occurs with the predictor venom component. Support is the number of transactions in which the desired venom component occurs.

Rules No.	Protein (Predictor)	Protein (Predicted)	Support	Confidence	Lift
1	SVMP_PI	LAAO	0.6	1	1.5
2	BPP, CRiSP	LAAO	0.6	1	1.5
3	CRiSP, CTL	LAAO	0.67	1	1.5
5	CRiSP, CTL	SVMP_PI	0.6	0.9	1.5
6	CTL	BPP	0.67	0.9	1.36
7	CTL	LAAO	0.67	0.9	1.36
8	CRiSP	LAAO	0.67	0.9	1.36
9	SVMP_PI	CTL	0.6	1	1.36
10	SVMP_PI	CRiSP	0.6	1	1.36
14	SVMP_PII, SVMP_PIII	CTL	0.53	1	1.36
15	CTL, SVMP_PII	SVMP_PIII	0.53	1	1.36
16	SVMP_PII, SVSP	SVMP_PIII	0.53	1	1.36
17	SVMP_PII, SVSP	CTL	0.53	1	1.36
18	BPP	LAAO	0.6	0.9	1.35
20	PLA2(Other), SVSP	SVMP_PIII	0.73	0.91	1.25
21	PLA2(Other), SVSP	CTL	0.73	0.91	1.25
22	PLA2(Other), SVSP	CRiSP	0.73	0.91	1.25
23	SVMP_PIII	CTL	0.67	0.9	1.23
25	SVMP_PIII	CRiSP	0.67	0.9	1.23
26	CRiSP	SVMP_PIII	0.67	0.9	1.24

Note: 5′-nucleotidase (5′-NT), bradykinin potentiate peptide (BPP), C-type lectins (CTL), cysteine-rich secretory protein (CRiSP), disintegrin (Dis), L-amino acid oxidase (LAAO), nerve growth factor (NGF), phosphodiesterase (PDE), phospholipase a_2_ (PLA_2_), snake venom metalloprotease (SVMP), and snake venom serine protease (SVSP).

**Table 4:** Venom components in *Sistrurus* genus.

Species	Venom Components	Reference
*S. catenatus*	3FTx, 5′-NT, BPP, CNP, CRiSP, CTL, Dis, GC, LAAO, MYO, NGF, PDE, PLA_2_ (CRTX, non-CRTX), PLB, Renin-like Aspartic Protease, SVMP P-I/II/III, SVSP, VEGF	[29,70,168,169,170,171,172,173]
*S. miliarius miliarius*	BPP, CRiSP, CTL, Dis, NGF, PLA_2_, SVMP P-I/III, SVMP-inhibitor, SVSP	[71]
*S. miliarius streckeri*	BPP, CRiSP, CTL, Dis, NGF, PLA_2_, SVMP P-I/III, SVMP-inhibitor, SVSP	[71]
*S. miliarius barbouri*	BPP, CNP, CRiSP, Dis, PLA_2_, SVMP P-I/III, SVSP	[29,30,70,71,173,174,175,176]

Note: three-finger toxin (3FTx), 5′-nucleotidase (5′-NT), bradykinin potentiate peptide (BPP), C-type lectins (CTL), natriuretic peptide type C (CNP), cysteine-rich secretory protein (CRiSP), crotoxin (CRTX), disintegrin (Dis), guanylyl cyclase (GC), hyaluronidase (Hya), L-amino acid oxidase (LAAO), myotoxin (MYO), nerve growth factor (NGF), phosphodiesterase (PDE), phospholipase a_2_ (PLA_2_), phospholipase b (PLB), snake venom metalloprotease (SVMP), snake venom serine protease (SVSP), and vascular endothelial growth factor (VEGF).

**Table 5:** Depictions of association rules between proteins expressed in *Sistrurus* venom. Lift signifies the correlation between different venom components. Confidence shows the percentage in which the predicted venom component occurs with the predictor venom component. Support is the number of transactions in which the desired venom component occurs.

Rules No.	Protein (Predictor)	Protein (Predicted)	Support	Confidence	Lift
1	SVSP	SVMP inhibitor	0.5	1	2
2	SVMP inhibitor	SVSP	0.5	1	2
3	SVSP	CTL	0.5	1	1.33
4	SVSP	NGF	0.5	1	1.33
5	SVMP inhibitor	CTL	0.5	1	1.33
6	SVMP inhibitor	NGF	0.5	1	1.33
7	CTL	NGF	0.75	1	1.33
8	NGF	CTL	0.75	1	1.33

Note: C-type lectins (CTL), nerve growth factor (NGF), snake venom metalloprotease (SVMP), snake venom metalloprotease inhibitor (SVMP inhibitor), snake venom serine protease (SVSP).

**Table 6:** Using maximum values of relative abundances of venom components, the association rules between proteins expressed in *Sistrurus* venom. Lift signifies the correlation between different venom components. Confidence shows the percentage in which the predicted venom component occurs with the predictor venom component. Support is the number of transactions in which the desired venom component occurs.

Rule No.	Protein Predictor	Protein (Predicted)	Support	Confidence	Lift
1	SVMP_PIII	SVMP_PI	0.5	1	2
3	SVMP_PIII	Vasoactive peptide	0.5	1	2
5	SVMP_PI	Vasoactive peptide	0.5	1	2
7	PLA2, CRTX	LAAO	0.5	1	1.5
8	SVMP_PIII	BPP	0.5	1	1.5
9	SVMP_PI	BPP	0.5	1	1.5
10	Vasoactive peptide	BPP	0.5	1	1.5
11	CNP	BPP	0.5	1	1.5
12	PLA2, CRTX	CTL	0.5	1	1.2
13	PLA2, CRTX	NGF	0.5	1	1.2
14	LAAO	CTL	0.67	1	1.2
15	LAAO	NGF	0.67	1	1.2
16	CTL	NGF	0.83	1	1.2

## Data Availability

Data sharing not applicable.

## Supplementary Materials

The following are available online at https://www.mdpi.com/article/10.3390/toxins13060372/s1, Table S1: Depictions of full association rules between proteins expressed in *Crotalus* venom using presence/absence data. Table S2: Depictions of all association rules between proteins expressed in *Crotalus* venom using relative abundance data.

## Abbreviations

The following abbreviations are used in this manuscript:

3FTx	Three-finger toxin
5′-NT	5′-nucleotidase
Achase	Acetylcholinesterase
ANP	Natriuretic peptide type A
ATPase	Adenosine triphosphatase
BIP	Bradykinin inhibitory peptide
BNP	Natriuretic peptide type B
BPP	Bradykinin potentiate peptide
CTL	C-type Lectins
CNP	Natriuretic peptide type C
CysProt	Cysteine protease
CRiSP	Cysteine-rich secretory protein
CA	Crotapotin
CRTX	Crotoxin
Dis	Disintegrin
EGF	Epidermal growth factor
FGF	Fibroblast growth factor
GC	Guanylyl cyclase
Hya	Hyaluronidase
Kazal	Kazal-type inhibitor
Kun	Kunitz-type inhibitor
LAAO	L-amino acid oxidase
MTX	Mojave toxin
MYO	Myotoxin
NGF	Nerve growth factor
OHA	Ohanin
PDE	Phosphodiesterase
PDGF	Platelet-derived growth factor
PLA_2_	Phospholipase A_2_
PLB	Phospholipase B
PLD	Phospholipase D
SVMP	Snake venom metalloprotease
SVSP	Snake venom serine protease
TLE	Thrombin-like enzyme
VEGF	Vascular endothelial growth factor
WAP	Waparin
N/A	No information available
*	Dataset used for generating hierarchical clusters
