# Supplementary material for: A Meta-Analysis of the Protein Components in Rattlesnake Venom
Source: Toxins (Basel). 2021 May 23;13(6):372. doi: 10.3390/toxins13060372 (PMC8224588; doi:10.3390/toxins13060372)
Supplement: Supplementary file 1 [file toxins-13-00372-s001.zip › toxins-1190419-supplementary.pdf]

Supplementary Materials: A Meta-Analysis of the Protein Components in Rattlesnake Venom

Anant Deshwal, Phuc Phan, Jyotishka Datta, Ragupathy Kannan and Suresh Kumar Thallapuranam

Table S1. Depictions of full association rules between proteins expressed in *Crotalus* venom.

Rules No.	Protein (Predictor)	Protein Predicted	Support	Confidence	Lift	Count
[4]	{SVMP_PI}	{LAAO}	0.571429	0.923077	1.140271	12
[5]	{SVMP_PI}	{SVSP}	0.571429	0.923077	0.969231	12
[6]	{SVMP_PI}	{Dis}	0.571429	0.923077	1.140271	12
[7]	{SVMP_PI}	{SVMP_PIII}	0.619048	1	1.05	13
[8]	{SVMP_PI}	{PLA2}	0.619048	1	1	13
[9]	{PDE}	{LAAO}	0.52381	1	1.235294	11
[10]	{PDE}	{Dis}	0.52381	1	1.235294	11
[11]	{PDE}	{SVMP_PIII}	0.52381	1	1.05	11
[12]	{PDE}	{PLA2}	0.52381	1	1	11
[13]	{Hya}	{SVMP_PIII}	0.52381	1	1.05	11
[14]	{Hya}	{PLA2}	0.52381	1	1	11
[15]	{BPP}	{CRiSP}	0.52381	1	1.4	11
[16]	{BPP}	{LAAO}	0.52381	1	1.235294	11
[17]	{BPP}	{Dis}	0.52381	1	1.235294	11
[18]	{BPP}	{SVMP_PIII}	0.52381	1	1.05	11
[19]	{BPP}	{PLA2}	0.52381	1	1	11
[20]	{CTL}	{CRiSP}	0.619048	0.928571	1.3	13
[21]	{CTL}	{LAAO}	0.666667	1	1.235294	14
[22]	{CTL}	{SVSP}	0.666667	1	1.05	14
[23]	{CTL}	{Dis}	0.666667	1	1.235294	14
[24]	{CTL}	{SVMP_PIII}	0.666667	1	1.05	14

[25]	{CTL}	{PLA2}	0.666667	1	1	14
[26]	{CRiSP}	{LAAO}	0.666667	0.933333	1.152941	14
[27]	{CRiSP}	{SVSP}	0.666667	0.933333	0.98	14
[28]	{CRiSP}	{Dis}	0.714286	1	1.235294	15
[29]	{CRiSP}	{SVMP_PIII}	0.714286	1	1.05	15
[30]	{CRiSP}	{PLA2}	0.714286	1	1	15
[31]	{LAAO}	{SVSP}	0.761905	0.941176	0.988235	16
[32]	{LAAO}	{Dis}	0.761905	0.941176	1.16263	16
[33]	{Dis}	{LAAO}	0.761905	0.941176	1.16263	16
[34]	{LAAO}	{SVMP_PIII}	0.761905	0.941176	0.988235	16
[35]	{LAAO}	{PLA2}	0.809524	1	1	17
[36]	{Dis}	{SVSP}	0.761905	0.941176	0.988235	16
[37]	{SVSP}	{SVMP_PIII}	0.904762	0.95	0.9975	19
[38]	{SVMP_PIII}	{SVSP}	0.904762	0.95	0.9975	19
[39]	{SVSP}	{PLA2}	0.952381	1	1	20
[40]	{PLA2}	{SVSP}	0.952381	0.952381	1	20
[41]	{Dis}	{SVMP_PIII}	0.809524	1	1.05	17
[42]	{Dis}	{PLA2}	0.809524	1	1	17
[43]	{SVMP_PIII}	{PLA2}	0.952381	1	1	20
[44]	{PLA2}	{SVMP_PIII}	0.952381	0.952381	1	20
[45]	{LAAO,SVMP_PI}	{SVSP}	0.52381	0.916667	0.9625	11
[46]	{SVMP_PI,SVSP}	{LAAO}	0.52381	0.916667	1.132353	11
[47]	{LAAO,SVMP_PI}	{Dis}	0.571429	1	1.235294	12
[48]	{Dis,SVMP_PI}	{LAAO}	0.571429	1	1.235294	12
[49]	{LAAO,SVMP_PI}	{SVMP_PIII}	0.571429	1	1.05	12
[50]	{SVMP_PI,SVMP_PIII}	{LAAO}	0.571429	0.923077	1.140271	12
[51]	{LAAO,SVMP_PI}	{PLA2}	0.571429	1	1	12
[52]	{PLA2,SVMP_PI}	{LAAO}	0.571429	0.923077	1.140271	12
[53]	{SVMP_PI,SVSP}	{Dis}	0.52381	0.916667	1.132353	11
[54]	{Dis,SVMP_PI}	{SVSP}	0.52381	0.916667	0.9625	11

[55]	{SVMP_PI,SVSP}	{SVMP_PIII}	0.571429	1	1.05	12
[56]	{SVMP_PI,SVMP_PIII}	{SVSP}	0.571429	0.923077	0.969231	12
[57]	{SVMP_PI,SVSP}	{PLA2}	0.571429	1	1	12
[58]	{PLA2,SVMP_PI}	{SVSP}	0.571429	0.923077	0.969231	12
[59]	{Dis,SVMP_PI}	{SVMP_PIII}	0.571429	1	1.05	12
[60]	{SVMP_PI,SVMP_PIII}	{Dis}	0.571429	0.923077	1.140271	12
[61]	{Dis,SVMP_PI}	{PLA2}	0.571429	1	1	12
[62]	{PLA2,SVMP_PI}	{Dis}	0.571429	0.923077	1.140271	12
[63]	{SVMP_PI,SVMP_PIII}	{PLA2}	0.619048	1	1	13
[64]	{PLA2,SVMP_PI}	{SVMP_PIII}	0.619048	1	1.05	13
[65]	{LAAO,PDE}	{Dis}	0.52381	1	1.235294	11
[66]	{Dis,PDE}	{LAAO}	0.52381	1	1.235294	11
[67]	{LAAO,PDE}	{SVMP_PIII}	0.52381	1	1.05	11
[68]	{PDE,SVMP_PIII}	{LAAO}	0.52381	1	1.235294	11
[69]	{LAAO,PDE}	{PLA2}	0.52381	1	1	11
[70]	{PDE,PLA2}	{LAAO}	0.52381	1	1.235294	11
[71]	{Dis,PDE}	{SVMP_PIII}	0.52381	1	1.05	11
[72]	{PDE,SVMP_PIII}	{Dis}	0.52381	1	1.235294	11
[73]	{Dis,PDE}	{PLA2}	0.52381	1	1	11
[74]	{PDE,PLA2}	{Dis}	0.52381	1	1.235294	11
[75]	{PDE,SVMP_PIII}	{PLA2}	0.52381	1	1	11
[76]	{PDE,PLA2}	{SVMP_PIII}	0.52381	1	1.05	11
[77]	{Hya,SVMP_PIII}	{PLA2}	0.52381	1	1	11
[78]	{Hya,PLA2}	{SVMP_PIII}	0.52381	1	1.05	11
[79]	{BPP,CRiSP}	{LAAO}	0.52381	1	1.235294	11
[80]	{BPP,LAAO}	{CRiSP}	0.52381	1	1.4	11
[81]	{BPP,CRiSP}	{Dis}	0.52381	1	1.235294	11
[82]	{BPP,Dis}	{CRiSP}	0.52381	1	1.4	11
[83]	{BPP,CRiSP}	{SVMP_PIII}	0.52381	1	1.05	11
[84]	{BPP,SVMP_PIII}	{CRiSP}	0.52381	1	1.4	11

[85]	{BPP,CRiSP}	{PLA2}	0.52381	1	1	11
[86]	{BPP,PLA2}	{CRiSP}	0.52381	1	1.4	11
[87]	{BPP,LAAO}	{Dis}	0.52381	1	1.235294	11
[88]	{BPP,Dis}	{LAAO}	0.52381	1	1.235294	11
[89]	{BPP,LAAO}	{SVMP_PIII}	0.52381	1	1.05	11
[90]	{BPP,SVMP_PIII}	{LAAO}	0.52381	1	1.235294	11
[91]	{BPP,LAAO}	{PLA2}	0.52381	1	1	11
[92]	{BPP,PLA2}	{LAAO}	0.52381	1	1.235294	11
[93]	{BPP,Dis}	{SVMP_PIII}	0.52381	1	1.05	11
[94]	{BPP,SVMP_PIII}	{Dis}	0.52381	1	1.235294	11
[95]	{BPP,Dis}	{PLA2}	0.52381	1	1	11
[96]	{BPP,PLA2}	{Dis}	0.52381	1	1.235294	11
[97]	{BPP,SVMP_PIII}	{PLA2}	0.52381	1	1	11
[98]	{BPP,PLA2}	{SVMP_PIII}	0.52381	1	1.05	11
[99]	{CRiSP,CTL}	{LAAO}	0.619048	1	1.235294	13
[100]	{CTL,LAAO}	{CRiSP}	0.619048	0.928571	1.3	13
[101]	{CRiSP,LAAO}	{CTL}	0.619048	0.928571	1.392857	13
[102]	{CRiSP,CTL}	{SVSP}	0.619048	1	1.05	13
[103]	{CTL,SVSP}	{CRiSP}	0.619048	0.928571	1.3	13
[104]	{CRiSP,SVSP}	{CTL}	0.619048	0.928571	1.392857	13
[105]	{CRiSP,CTL}	{Dis}	0.619048	1	1.235294	13
[106]	{CTL,Dis}	{CRiSP}	0.619048	0.928571	1.3	13
[107]	{CRiSP,CTL}	{SVMP_PIII}	0.619048	1	1.05	13
[108]	{CTL,SVMP_PIII}	{CRiSP}	0.619048	0.928571	1.3	13
[109]	{CRiSP,CTL}	{PLA2}	0.619048	1	1	13
[110]	{CTL,PLA2}	{CRiSP}	0.619048	0.928571	1.3	13
[111]	{CTL,LAAO}	{SVSP}	0.666667	1	1.05	14
[112]	{CTL,SVSP}	{LAAO}	0.666667	1	1.235294	14
[113]	{CTL,LAAO}	{Dis}	0.666667	1	1.235294	14
[114]	{CTL,Dis}	{LAAO}	0.666667	1	1.235294	14

[115]	{CTL,LAAO}	{SVMP_PIII}	0.666667	1	1.05	14
[116]	{CTL,SVMP_PIII}	{LAAO}	0.666667	1	1.235294	14
[117]	{CTL,LAAO}	{PLA2}	0.666667	1	1	14
[118]	{CTL,PLA2}	{LAAO}	0.666667	1	1.235294	14
[119]	{CTL,SVSP}	{Dis}	0.666667	1	1.235294	14
[120]	{CTL,Dis}	{SVSP}	0.666667	1	1.05	14
[121]	{CTL,SVSP}	{SVMP_PIII}	0.666667	1	1.05	14
[122]	{CTL,SVMP_PIII}	{SVSP}	0.666667	1	1.05	14
[123]	{CTL,SVSP}	{PLA2}	0.666667	1	1	14
[124]	{CTL,PLA2}	{SVSP}	0.666667	1	1.05	14
[125]	{CTL,Dis}	{SVMP_PIII}	0.666667	1	1.05	14
[126]	{CTL,SVMP_PIII}	{Dis}	0.666667	1	1.235294	14
[127]	{CTL,Dis}	{PLA2}	0.666667	1	1	14
[128]	{CTL,PLA2}	{Dis}	0.666667	1	1.235294	14
[129]	{CTL,SVMP_PIII}	{PLA2}	0.666667	1	1	14
[130]	{CTL,PLA2}	{SVMP_PIII}	0.666667	1	1.05	14
[131]	{CRiSP,LAAO}	{SVSP}	0.619048	0.928571	0.975	13
[132]	{CRiSP,SVSP}	{LAAO}	0.619048	0.928571	1.147059	13
[133]	{CRiSP,LAAO}	{Dis}	0.666667	1	1.235294	14
[134]	{CRiSP,Dis}	{LAAO}	0.666667	0.933333	1.152941	14
[135]	{CRiSP,LAAO}	{SVMP_PIII}	0.666667	1	1.05	14
[136]	{CRiSP,SVMP_PIII}	{LAAO}	0.666667	0.933333	1.152941	14
[137]	{CRiSP,LAAO}	{PLA2}	0.666667	1	1	14
[138]	{CRiSP,PLA2}	{LAAO}	0.666667	0.933333	1.152941	14
[139]	{CRiSP,SVSP}	{Dis}	0.666667	1	1.235294	14
[140]	{CRiSP,Dis}	{SVSP}	0.666667	0.933333	0.98	14
[141]	{CRiSP,SVSP}	{SVMP_PIII}	0.666667	1	1.05	14
[142]	{CRiSP,SVMP_PIII}	{SVSP}	0.666667	0.933333	0.98	14
[143]	{CRiSP,SVSP}	{PLA2}	0.666667	1	1	14
[144]	{CRiSP,PLA2}	{SVSP}	0.666667	0.933333	0.98	14

[145]	{CRiSP,Dis}	{SVMP_PIII}	0.714286	1	1.05	15
[146]	{CRiSP,SVMP_PIII}	{Dis}	0.714286	1	1.235294	15
[147]	{CRiSP,Dis}	{PLA2}	0.714286	1	1	15
[148]	{CRiSP,PLA2}	{Dis}	0.714286	1	1.235294	15
[149]	{CRiSP,SVMP_PIII}	{PLA2}	0.714286	1	1	15
[150]	{CRiSP,PLA2}	{SVMP_PIII}	0.714286	1	1.05	15
[151]	{LAAO,SVSP}	{Dis}	0.714286	0.9375	1.158088	15
[152]	{Dis,LAAO}	{SVSP}	0.714286	0.9375	0.984375	15
[153]	{Dis,SVSP}	{LAAO}	0.714286	0.9375	1.158088	15
[154]	{LAAO,SVSP}	{SVMP_PIII}	0.714286	0.9375	0.984375	15
[155]	{LAAO,SVMP_PIII}	{SVSP}	0.714286	0.9375	0.984375	15
[156]	{LAAO,SVSP}	{PLA2}	0.761905	1	1	16
[157]	{LAAO,PLA2}	{SVSP}	0.761905	0.941176	0.988235	16
[158]	{Dis,LAAO}	{SVMP_PIII}	0.761905	1	1.05	16
[159]	{LAAO,SVMP_PIII}	{Dis}	0.761905	1	1.235294	16
[160]	{Dis,SVMP_PIII}	{LAAO}	0.761905	0.941176	1.16263	16
[161]	{Dis,LAAO}	{PLA2}	0.761905	1	1	16
[162]	{LAAO,PLA2}	{Dis}	0.761905	0.941176	1.16263	16
[163]	{Dis,PLA2}	{LAAO}	0.761905	0.941176	1.16263	16
[164]	{LAAO,SVMP_PIII}	{PLA2}	0.761905	1	1	16
[165]	{LAAO,PLA2}	{SVMP_PIII}	0.761905	0.941176	0.988235	16
[166]	{Dis,SVSP}	{SVMP_PIII}	0.761905	1	1.05	16
[167]	{Dis,SVMP_PIII}	{SVSP}	0.761905	0.941176	0.988235	16
[168]	{Dis,SVSP}	{PLA2}	0.761905	1	1	16
[169]	{Dis,PLA2}	{SVSP}	0.761905	0.941176	0.988235	16
[170]	{SVMP_PIII,SVSP}	{PLA2}	0.904762	1	1	19
[171]	{PLA2,SVSP}	{SVMP_PIII}	0.904762	0.95	0.9975	19
[172]	{PLA2,SVMP_PIII}	{SVSP}	0.904762	0.95	0.9975	19
[173]	{Dis,SVMP_PIII}	{PLA2}	0.809524	1	1	17
[174]	{Dis,PLA2}	{SVMP_PIII}	0.809524	1	1.05	17

[175]	{LAAO,SVMP_PI,SVSP}	{Dis}	0.52381	1	1.235294	11
[176]	{Dis,LAAO,SVMP_PI}	{SVSP}	0.52381	0.916667	0.9625	11
[177]	{Dis,SVMP_PI,SVSP}	{LAAO}	0.52381	1	1.235294	11
[178]	{LAAO,SVMP_PI,SVSP}	{SVMP_PIII}	0.52381	1	1.05	11
[179]	{LAAO,SVMP_PI,SVMP_PIII}	{SVSP}	0.52381	0.916667	0.9625	11
[180]	{SVMP_PI,SVMP_PIII,SVSP}	{LAAO}	0.52381	0.916667	1.132353	11
[181]	{LAAO,SVMP_PI,SVSP}	{PLA2}	0.52381	1	1	11
[182]	{LAAO,PLA2,SVMP_PI}	{SVSP}	0.52381	0.916667	0.9625	11
[183]	{PLA2,SVMP_PI,SVSP}	{LAAO}	0.52381	0.916667	1.132353	11
[184]	{Dis,LAAO,SVMP_PI}	{SVMP_PIII}	0.571429	1	1.05	12
[185]	{LAAO,SVMP_PI,SVMP_PIII}	{Dis}	0.571429	1	1.235294	12
[186]	{Dis,SVMP_PI,SVMP_PIII}	{LAAO}	0.571429	1	1.235294	12
[187]	{Dis,LAAO,SVMP_PI}	{PLA2}	0.571429	1	1	12
[188]	{LAAO,PLA2,SVMP_PI}	{Dis}	0.571429	1	1.235294	12
[189]	{Dis,PLA2,SVMP_PI}	{LAAO}	0.571429	1	1.235294	12
[190]	{LAAO,SVMP_PI,SVMP_PIII}	{PLA2}	0.571429	1	1	12
[191]	{LAAO,PLA2,SVMP_PI}	{SVMP_PIII}	0.571429	1	1.05	12
[192]	{PLA2,SVMP_PI,SVMP_PIII}	{LAAO}	0.571429	0.923077	1.140271	12
[193]	{Dis,SVMP_PI,SVSP}	{SVMP_PIII}	0.52381	1	1.05	11
[194]	{SVMP_PI,SVMP_PIII,SVSP}	{Dis}	0.52381	0.916667	1.132353	11
[195]	{Dis,SVMP_PI,SVMP_PIII}	{SVSP}	0.52381	0.916667	0.9625	11
[196]	{Dis,SVMP_PI,SVSP}	{PLA2}	0.52381	1	1	11
[197]	{PLA2,SVMP_PI,SVSP}	{Dis}	0.52381	0.916667	1.132353	11
[198]	{Dis,PLA2,SVMP_PI}	{SVSP}	0.52381	0.916667	0.9625	11
[199]	{SVMP_PI,SVMP_PIII,SVSP}	{PLA2}	0.571429	1	1	12
[200]	{PLA2,SVMP_PI,SVSP}	{SVMP_PIII}	0.571429	1	1.05	12
[201]	{PLA2,SVMP_PI,SVMP_PIII}	{SVSP}	0.571429	0.923077	0.969231	12
[202]	{Dis,SVMP_PI,SVMP_PIII}	{PLA2}	0.571429	1	1	12
[203]	{Dis,PLA2,SVMP_PI}	{SVMP_PIII}	0.571429	1	1.05	12
[204]	{PLA2,SVMP_PI,SVMP_PIII}	{Dis}	0.571429	0.923077	1.140271	12

[205]	{Dis,LAAO,PDE}	{SVMP_PIII}	0.52381	1	1.05	11
[206]	{LAAO,PDE,SVMP_PIII}	{Dis}	0.52381	1	1.235294	11
[207]	{Dis,PDE,SVMP_PIII}	{LAAO}	0.52381	1	1.235294	11
[208]	{Dis,LAAO,PDE}	{PLA2}	0.52381	1	1	11
[209]	{LAAO,PDE,PLA2}	{Dis}	0.52381	1	1.235294	11
[210]	{Dis,PDE,PLA2}	{LAAO}	0.52381	1	1.235294	11
[211]	{LAAO,PDE,SVMP_PIII}	{PLA2}	0.52381	1	1	11
[212]	{LAAO,PDE,PLA2}	{SVMP_PIII}	0.52381	1	1.05	11
[213]	{PDE,PLA2,SVMP_PIII}	{LAAO}	0.52381	1	1.235294	11
[214]	{Dis,PDE,SVMP_PIII}	{PLA2}	0.52381	1	1	11
[215]	{Dis,PDE,PLA2}	{SVMP_PIII}	0.52381	1	1.05	11
[216]	{PDE,PLA2,SVMP_PIII}	{Dis}	0.52381	1	1.235294	11
[217]	{BPP,CRiSP,LAAO}	{Dis}	0.52381	1	1.235294	11
[218]	{BPP,CRiSP,Dis}	{LAAO}	0.52381	1	1.235294	11
[219]	{BPP,Dis,LAAO}	{CRiSP}	0.52381	1	1.4	11
[220]	{BPP,CRiSP,LAAO}	{SVMP_PIII}	0.52381	1	1.05	11
[221]	{BPP,CRiSP,SVMP_PIII}	{LAAO}	0.52381	1	1.235294	11
[222]	{BPP,LAAO,SVMP_PIII}	{CRiSP}	0.52381	1	1.4	11
[223]	{BPP,CRiSP,LAAO}	{PLA2}	0.52381	1	1	11
[224]	{BPP,CRiSP,PLA2}	{LAAO}	0.52381	1	1.235294	11
[225]	{BPP,LAAO,PLA2}	{CRiSP}	0.52381	1	1.4	11
[226]	{BPP,CRiSP,Dis}	{SVMP_PIII}	0.52381	1	1.05	11
[227]	{BPP,CRiSP,SVMP_PIII}	{Dis}	0.52381	1	1.235294	11
[228]	{BPP,Dis,SVMP_PIII}	{CRiSP}	0.52381	1	1.4	11
[229]	{BPP,CRiSP,Dis}	{PLA2}	0.52381	1	1	11
[230]	{BPP,CRiSP,PLA2}	{Dis}	0.52381	1	1.235294	11
[231]	{BPP,Dis,PLA2}	{CRiSP}	0.52381	1	1.4	11
[232]	{BPP,CRiSP,SVMP_PIII}	{PLA2}	0.52381	1	1	11
[233]	{BPP,CRiSP,PLA2}	{SVMP_PIII}	0.52381	1	1.05	11
[234]	{BPP,PLA2,SVMP_PIII}	{CRiSP}	0.52381	1	1.4	11

[235]	{BPP,Dis,LAAO}	{SVMP_PIII}	0.52381	1	1.05	11
[236]	{BPP,LAAO,SVMP_PIII}	{Dis}	0.52381	1	1.235294	11
[237]	{BPP,Dis,SVMP_PIII}	{LAAO}	0.52381	1	1.235294	11
[238]	{BPP,Dis,LAAO}	{PLA2}	0.52381	1	1	11
[239]	{BPP,LAAO,PLA2}	{Dis}	0.52381	1	1.235294	11
[240]	{BPP,Dis,PLA2}	{LAAO}	0.52381	1	1.235294	11
[241]	{BPP,LAAO,SVMP_PIII}	{PLA2}	0.52381	1	1	11
[242]	{BPP,LAAO,PLA2}	{SVMP_PIII}	0.52381	1	1.05	11
[243]	{BPP,PLA2,SVMP_PIII}	{LAAO}	0.52381	1	1.235294	11
[244]	{BPP,Dis,SVMP_PIII}	{PLA2}	0.52381	1	1	11
[245]	{BPP,Dis,PLA2}	{SVMP_PIII}	0.52381	1	1.05	11
[246]	{BPP,PLA2,SVMP_PIII}	{Dis}	0.52381	1	1.235294	11
[247]	{CRiSP,CTL,LAAO}	{SVSP}	0.619048	1	1.05	13
[248]	{CRiSP,CTL,SVSP}	{LAAO}	0.619048	1	1.235294	13
[249]	{CTL,LAAO,SVSP}	{CRiSP}	0.619048	0.928571	1.3	13
[250]	{CRiSP,LAAO,SVSP}	{CTL}	0.619048	1	1.5	13
[251]	{CRiSP,CTL,LAAO}	{Dis}	0.619048	1	1.235294	13
[252]	{CRiSP,CTL,Dis}	{LAAO}	0.619048	1	1.235294	13
[253]	{CTL,Dis,LAAO}	{CRiSP}	0.619048	0.928571	1.3	13
[254]	{CRiSP,Dis,LAAO}	{CTL}	0.619048	0.928571	1.392857	13
[255]	{CRiSP,CTL,LAAO}	{SVMP_PIII}	0.619048	1	1.05	13
[256]	{CRiSP,CTL,SVMP_PIII}	{LAAO}	0.619048	1	1.235294	13
[257]	{CTL,LAAO,SVMP_PIII}	{CRiSP}	0.619048	0.928571	1.3	13
[258]	{CRiSP,LAAO,SVMP_PIII}	{CTL}	0.619048	0.928571	1.392857	13
[259]	{CRiSP,CTL,LAAO}	{PLA2}	0.619048	1	1	13
[260]	{CRiSP,CTL,PLA2}	{LAAO}	0.619048	1	1.235294	13
[261]	{CTL,LAAO,PLA2}	{CRiSP}	0.619048	0.928571	1.3	13
[262]	{CRiSP,LAAO,PLA2}	{CTL}	0.619048	0.928571	1.392857	13
[263]	{CRiSP,CTL,SVSP}	{Dis}	0.619048	1	1.235294	13
[264]	{CRiSP,CTL,Dis}	{SVSP}	0.619048	1	1.05	13

[265]	{CTL,Dis,SVSP}	{CRiSP}	0.619048	0.928571	1.3	13
[266]	{CRiSP,Dis,SVSP}	{CTL}	0.619048	0.928571	1.392857	13
[267]	{CRiSP,CTL,SVSP}	{SVMP_PIII}	0.619048	1	1.05	13
[268]	{CRiSP,CTL,SVMP_PIII}	{SVSP}	0.619048	1	1.05	13
[269]	{CTL,SVMP_PIII,SVSP}	{CRiSP}	0.619048	0.928571	1.3	13
[270]	{CRiSP,SVMP_PIII,SVSP}	{CTL}	0.619048	0.928571	1.392857	13
[271]	{CRiSP,CTL,SVSP}	{PLA2}	0.619048	1	1	13
[272]	{CRiSP,CTL,PLA2}	{SVSP}	0.619048	1	1.05	13
[273]	{CTL,PLA2,SVSP}	{CRiSP}	0.619048	0.928571	1.3	13
[274]	{CRiSP,PLA2,SVSP}	{CTL}	0.619048	0.928571	1.392857	13
[275]	{CRiSP,CTL,Dis}	{SVMP_PIII}	0.619048	1	1.05	13
[276]	{CRiSP,CTL,SVMP_PIII}	{Dis}	0.619048	1	1.235294	13
[277]	{CTL,Dis,SVMP_PIII}	{CRiSP}	0.619048	0.928571	1.3	13
[278]	{CRiSP,CTL,Dis}	{PLA2}	0.619048	1	1	13
[279]	{CRiSP,CTL,PLA2}	{Dis}	0.619048	1	1.235294	13
[280]	{CTL,Dis,PLA2}	{CRiSP}	0.619048	0.928571	1.3	13
[281]	{CRiSP,CTL,SVMP_PIII}	{PLA2}	0.619048	1	1	13
[282]	{CRiSP,CTL,PLA2}	{SVMP_PIII}	0.619048	1	1.05	13
[283]	{CTL,PLA2,SVMP_PIII}	{CRiSP}	0.619048	0.928571	1.3	13
[284]	{CTL,LAAO,SVSP}	{Dis}	0.666667	1	1.235294	14
[285]	{CTL,Dis,LAAO}	{SVSP}	0.666667	1	1.05	14
[286]	{CTL,Dis,SVSP}	{LAAO}	0.666667	1	1.235294	14
[287]	{Dis,LAAO,SVSP}	{CTL}	0.666667	0.933333	1.4	14
[288]	{CTL,LAAO,SVSP}	{SVMP_PIII}	0.666667	1	1.05	14
[289]	{CTL,LAAO,SVMP_PIII}	{SVSP}	0.666667	1	1.05	14
[290]	{CTL,SVMP_PIII,SVSP}	{LAAO}	0.666667	1	1.235294	14
[291]	{LAAO,SVMP_PIII,SVSP}	{CTL}	0.666667	0.933333	1.4	14
[292]	{CTL,LAAO,SVSP}	{PLA2}	0.666667	1	1	14
[293]	{CTL,LAAO,PLA2}	{SVSP}	0.666667	1	1.05	14
[294]	{CTL,PLA2,SVSP}	{LAAO}	0.666667	1	1.235294	14

[295]	{CTL,Dis,LAAO}	{SVMP_PIII}	0.666667	1	1.05	14
[296]	{CTL,LAAO,SVMP_PIII}	{Dis}	0.666667	1	1.235294	14
[297]	{CTL,Dis,SVMP_PIII}	{LAAO}	0.666667	1	1.235294	14
[298]	{CTL,Dis,LAAO}	{PLA2}	0.666667	1	1	14
[299]	{CTL,LAAO,PLA2}	{Dis}	0.666667	1	1.235294	14
[300]	{CTL,Dis,PLA2}	{LAAO}	0.666667	1	1.235294	14
[301]	{CTL,LAAO,SVMP_PIII}	{PLA2}	0.666667	1	1	14
[302]	{CTL,LAAO,PLA2}	{SVMP_PIII}	0.666667	1	1.05	14
[303]	{CTL,PLA2,SVMP_PIII}	{LAAO}	0.666667	1	1.235294	14
[304]	{CTL,Dis,SVSP}	{SVMP_PIII}	0.666667	1	1.05	14
[305]	{CTL,SVMP_PIII,SVSP}	{Dis}	0.666667	1	1.235294	14
[306]	{CTL,Dis,SVMP_PIII}	{SVSP}	0.666667	1	1.05	14
[307]	{CTL,Dis,SVSP}	{PLA2}	0.666667	1	1	14
[308]	{CTL,PLA2,SVSP}	{Dis}	0.666667	1	1.235294	14
[309]	{CTL,Dis,PLA2}	{SVSP}	0.666667	1	1.05	14
[310]	{CTL,SVMP_PIII,SVSP}	{PLA2}	0.666667	1	1	14
[311]	{CTL,PLA2,SVSP}	{SVMP_PIII}	0.666667	1	1.05	14
[312]	{CTL,PLA2,SVMP_PIII}	{SVSP}	0.666667	1	1.05	14
[313]	{CTL,Dis,SVMP_PIII}	{PLA2}	0.666667	1	1	14
[314]	{CTL,Dis,PLA2}	{SVMP_PIII}	0.666667	1	1.05	14
[315]	{CTL,PLA2,SVMP_PIII}	{Dis}	0.666667	1	1.235294	14
[316]	{CRiSP,LAAO,SVSP}	{Dis}	0.619048	1	1.235294	13
[317]	{CRiSP,Dis,LAAO}	{SVSP}	0.619048	0.928571	0.975	13
[318]	{CRiSP,Dis,SVSP}	{LAAO}	0.619048	0.928571	1.147059	13
[319]	{CRiSP,LAAO,SVSP}	{SVMP_PIII}	0.619048	1	1.05	13
[320]	{CRiSP,LAAO,SVMP_PIII}	{SVSP}	0.619048	0.928571	0.975	13
[321]	{CRiSP,SVMP_PIII,SVSP}	{LAAO}	0.619048	0.928571	1.147059	13
[322]	{CRiSP,LAAO,SVSP}	{PLA2}	0.619048	1	1	13
[323]	{CRiSP,LAAO,PLA2}	{SVSP}	0.619048	0.928571	0.975	13
[324]	{CRiSP,PLA2,SVSP}	{LAAO}	0.619048	0.928571	1.147059	13

[325]	{CRiSP,Dis,LAAO}	{SVMP_PIII}	0.666667	1	1.05	14
[326]	{CRiSP,LAAO,SVMP_PIII}	{Dis}	0.666667	1	1.235294	14
[327]	{CRiSP,Dis,SVMP_PIII}	{LAAO}	0.666667	0.933333	1.152941	14
[328]	{CRiSP,Dis,LAAO}	{PLA2}	0.666667	1	1	14
[329]	{CRiSP,LAAO,PLA2}	{Dis}	0.666667	1	1.235294	14
[330]	{CRiSP,Dis,PLA2}	{LAAO}	0.666667	0.933333	1.152941	14
[331]	{CRiSP,LAAO,SVMP_PIII}	{PLA2}	0.666667	1	1	14
[332]	{CRiSP,LAAO,PLA2}	{SVMP_PIII}	0.666667	1	1.05	14
[333]	{CRiSP,PLA2,SVMP_PIII}	{LAAO}	0.666667	0.933333	1.152941	14
[334]	{CRiSP,Dis,SVSP}	{SVMP_PIII}	0.666667	1	1.05	14
[335]	{CRiSP,SVMP_PIII,SVSP}	{Dis}	0.666667	1	1.235294	14
[336]	{CRiSP,Dis,SVMP_PIII}	{SVSP}	0.666667	0.933333	0.98	14
[337]	{CRiSP,Dis,SVSP}	{PLA2}	0.666667	1	1	14
[338]	{CRiSP,PLA2,SVSP}	{Dis}	0.666667	1	1.235294	14
[339]	{CRiSP,Dis,PLA2}	{SVSP}	0.666667	0.933333	0.98	14
[340]	{CRiSP,SVMP_PIII,SVSP}	{PLA2}	0.666667	1	1	14
[341]	{CRiSP,PLA2,SVSP}	{SVMP_PIII}	0.666667	1	1.05	14
[342]	{CRiSP,PLA2,SVMP_PIII}	{SVSP}	0.666667	0.933333	0.98	14
[343]	{CRiSP,Dis,SVMP_PIII}	{PLA2}	0.714286	1	1	15
[344]	{CRiSP,Dis,PLA2}	{SVMP_PIII}	0.714286	1	1.05	15
[345]	{CRiSP,PLA2,SVMP_PIII}	{Dis}	0.714286	1	1.235294	15
[346]	{Dis,LAAO,SVSP}	{SVMP_PIII}	0.714286	1	1.05	15
[347]	{LAAO,SVMP_PIII,SVSP}	{Dis}	0.714286	1	1.235294	15
[348]	{Dis,LAAO,SVMP_PIII}	{SVSP}	0.714286	0.9375	0.984375	15
[349]	{Dis,SVMP_PIII,SVSP}	{LAAO}	0.714286	0.9375	1.158088	15
[350]	{Dis,LAAO,SVSP}	{PLA2}	0.714286	1	1	15
[351]	{LAAO,PLA2,SVSP}	{Dis}	0.714286	0.9375	1.158088	15
[352]	{Dis,LAAO,PLA2}	{SVSP}	0.714286	0.9375	0.984375	15
[353]	{Dis,PLA2,SVSP}	{LAAO}	0.714286	0.9375	1.158088	15
[354]	{LAAO,SVMP_PIII,SVSP}	{PLA2}	0.714286	1	1	15

[355]	{LAAO,PLA2,SVSP}	{SVMP_PIII}	0.714286	0.9375	0.984375	15
[356]	{LAAO,PLA2,SVMP_PIII}	{SVSP}	0.714286	0.9375	0.984375	15
[357]	{Dis,LAAO,SVMP_PIII}	{PLA2}	0.761905	1	1	16
[358]	{Dis,LAAO,PLA2}	{SVMP_PIII}	0.761905	1	1.05	16
[359]	{LAAO,PLA2,SVMP_PIII}	{Dis}	0.761905	1	1.235294	16
[360]	{Dis,PLA2,SVMP_PIII}	{LAAO}	0.761905	0.941176	1.16263	16
[361]	{Dis,SVMP_PIII,SVSP}	{PLA2}	0.761905	1	1	16
[362]	{Dis,PLA2,SVSP}	{SVMP_PIII}	0.761905	1	1.05	16
[363]	{Dis,PLA2,SVMP_PIII}	{SVSP}	0.761905	0.941176	0.988235	16
[364]	{Dis,LAAO,SVMP_PI,SVSP}	{SVMP_PIII}	0.52381	1	1.05	11
[365]	{LAAO,SVMP_PI,SVMP_PIII,SVSP}	{Dis}	0.52381	1	1.235294	11
[366]	{Dis,LAAO,SVMP_PI,SVMP_PIII}	{SVSP}	0.52381	0.916667	0.9625	11
[367]	{Dis,SVMP_PI,SVMP_PIII,SVSP}	{LAAO}	0.52381	1	1.235294	11
[368]	{Dis,LAAO,SVMP_PI,SVSP}	{PLA2}	0.52381	1	1	11
[369]	{LAAO,PLA2,SVMP_PI,SVSP}	{Dis}	0.52381	1	1.235294	11
[370]	{Dis,LAAO,PLA2,SVMP_PI}	{SVSP}	0.52381	0.916667	0.9625	11
[371]	{Dis,PLA2,SVMP_PI,SVSP}	{LAAO}	0.52381	1	1.235294	11
[372]	{LAAO,SVMP_PI,SVMP_PIII,SVSP}	{PLA2}	0.52381	1	1	11
[373]	{LAAO,PLA2,SVMP_PI,SVSP}	{SVMP_PIII}	0.52381	1	1.05	11
[374]	{LAAO,PLA2,SVMP_PI,SVMP_PIII}	{SVSP}	0.52381	0.916667	0.9625	11
[375]	{PLA2,SVMP_PI,SVMP_PIII,SVSP}	{LAAO}	0.52381	0.916667	1.132353	11
[376]	{Dis,LAAO,SVMP_PI,SVMP_PIII}	{PLA2}	0.571429	1	1	12
[377]	{Dis,LAAO,PLA2,SVMP_PI}	{SVMP_PIII}	0.571429	1	1.05	12
[378]	{LAAO,PLA2,SVMP_PI,SVMP_PIII}	{Dis}	0.571429	1	1.235294	12
[379]	{Dis,PLA2,SVMP_PI,SVMP_PIII}	{LAAO}	0.571429	1	1.235294	12
[380]	{Dis,SVMP_PI,SVMP_PIII,SVSP}	{PLA2}	0.52381	1	1	11
[381]	{Dis,PLA2,SVMP_PI,SVSP}	{SVMP_PIII}	0.52381	1	1.05	11
[382]	{PLA2,SVMP_PI,SVMP_PIII,SVSP}	{Dis}	0.52381	0.916667	1.132353	11
[383]	{Dis,PLA2,SVMP_PI,SVMP_PIII}	{SVSP}	0.52381	0.916667	0.9625	11
[384]	{Dis,LAAO,PDE,SVMP_PIII}	{PLA2}	0.52381	1	1	11

[385]	{Dis,LAAO,PDE,PLA2}	{SVMP_PIII}	0.52381	1	1.05	11
[386]	{LAAO,PDE,PLA2,SVMP_PIII}	{Dis}	0.52381	1	1.235294	11
[387]	{Dis,PDE,PLA2,SVMP_PIII}	{LAAO}	0.52381	1	1.235294	11
[388]	{BPP,CRiSP,Dis,LAAO}	{SVMP_PIII}	0.52381	1	1.05	11
[389]	{BPP,CRiSP,LAAO,SVMP_PIII}	{Dis}	0.52381	1	1.235294	11
[390]	{BPP,CRiSP,Dis,SVMP_PIII}	{LAAO}	0.52381	1	1.235294	11
[391]	{BPP,Dis,LAAO,SVMP_PIII}	{CRiSP}	0.52381	1	1.4	11
[392]	{BPP,CRiSP,Dis,LAAO}	{PLA2}	0.52381	1	1	11
[393]	{BPP,CRiSP,LAAO,PLA2}	{Dis}	0.52381	1	1.235294	11
[394]	{BPP,CRiSP,Dis,PLA2}	{LAAO}	0.52381	1	1.235294	11
[395]	{BPP,Dis,LAAO,PLA2}	{CRiSP}	0.52381	1	1.4	11
[396]	{BPP,CRiSP,LAAO,SVMP_PIII}	{PLA2}	0.52381	1	1	11
[397]	{BPP,CRiSP,LAAO,PLA2}	{SVMP_PIII}	0.52381	1	1.05	11
[398]	{BPP,CRiSP,PLA2,SVMP_PIII}	{LAAO}	0.52381	1	1.235294	11
[399]	{BPP,LAAO,PLA2,SVMP_PIII}	{CRiSP}	0.52381	1	1.4	11
[400]	{BPP,CRiSP,Dis,SVMP_PIII}	{PLA2}	0.52381	1	1	11
[401]	{BPP,CRiSP,Dis,PLA2}	{SVMP_PIII}	0.52381	1	1.05	11
[402]	{BPP,CRiSP,PLA2,SVMP_PIII}	{Dis}	0.52381	1	1.235294	11
[403]	{BPP,Dis,PLA2,SVMP_PIII}	{CRiSP}	0.52381	1	1.4	11
[404]	{BPP,Dis,LAAO,SVMP_PIII}	{PLA2}	0.52381	1	1	11
[405]	{BPP,Dis,LAAO,PLA2}	{SVMP_PIII}	0.52381	1	1.05	11
[406]	{BPP,LAAO,PLA2,SVMP_PIII}	{Dis}	0.52381	1	1.235294	11
[407]	{BPP,Dis,PLA2,SVMP_PIII}	{LAAO}	0.52381	1	1.235294	11
[408]	{CRiSP,CTL,LAAO,SVSP}	{Dis}	0.619048	1	1.235294	13
[409]	{CRiSP,CTL,Dis,LAAO}	{SVSP}	0.619048	1	1.05	13
[410]	{CRiSP,CTL,Dis,SVSP}	{LAAO}	0.619048	1	1.235294	13
[411]	{CTL,Dis,LAAO,SVSP}	{CRiSP}	0.619048	0.928571	1.3	13
[412]	{CRiSP,Dis,LAAO,SVSP}	{CTL}	0.619048	1	1.5	13
[413]	{CRiSP,CTL,LAAO,SVSP}	{SVMP_PIII}	0.619048	1	1.05	13
[414]	{CRiSP,CTL,LAAO,SVMP_PIII}	{SVSP}	0.619048	1	1.05	13

[415]	{CRiSP,CTL,SVMP_PIII,SVSP}	{LAAO}	0.619048	1	1.235294	13
[416]	{CTL,LAAO,SVMP_PIII,SVSP}	{CRiSP}	0.619048	0.928571	1.3	13
[417]	{CRiSP,LAAO,SVMP_PIII,SVSP}	{CTL}	0.619048	1	1.5	13
[418]	{CRiSP,CTL,LAAO,SVSP}	{PLA2}	0.619048	1	1	13
[419]	{CRiSP,CTL,LAAO,PLA2}	{SVSP}	0.619048	1	1.05	13
[420]	{CRiSP,CTL,PLA2,SVSP}	{LAAO}	0.619048	1	1.235294	13
[421]	{CTL,LAAO,PLA2,SVSP}	{CRiSP}	0.619048	0.928571	1.3	13
[422]	{CRiSP,LAAO,PLA2,SVSP}	{CTL}	0.619048	1	1.5	13
[423]	{CRiSP,CTL,Dis,LAAO}	{SVMP_PIII}	0.619048	1	1.05	13
[424]	{CRiSP,CTL,LAAO,SVMP_PIII}	{Dis}	0.619048	1	1.235294	13
[425]	{CRiSP,CTL,Dis,SVMP_PIII}	{LAAO}	0.619048	1	1.235294	13
[426]	{CTL,Dis,LAAO,SVMP_PIII}	{CRiSP}	0.619048	0.928571	1.3	13
[427]	{CRiSP,Dis,LAAO,SVMP_PIII}	{CTL}	0.619048	0.928571	1.392857	13
[428]	{CRiSP,CTL,Dis,LAAO}	{PLA2}	0.619048	1	1	13
[429]	{CRiSP,CTL,LAAO,PLA2}	{Dis}	0.619048	1	1.235294	13
[430]	{CRiSP,CTL,Dis,PLA2}	{LAAO}	0.619048	1	1.235294	13
[431]	{CTL,Dis,LAAO,PLA2}	{CRiSP}	0.619048	0.928571	1.3	13
[432]	{CRiSP,Dis,LAAO,PLA2}	{CTL}	0.619048	0.928571	1.392857	13
[433]	{CRiSP,CTL,LAAO,SVMP_PIII}	{PLA2}	0.619048	1	1	13
[434]	{CRiSP,CTL,LAAO,PLA2}	{SVMP_PIII}	0.619048	1	1.05	13
[435]	{CRiSP,CTL,PLA2,SVMP_PIII}	{LAAO}	0.619048	1	1.235294	13
[436]	{CTL,LAAO,PLA2,SVMP_PIII}	{CRiSP}	0.619048	0.928571	1.3	13
[437]	{CRiSP,LAAO,PLA2,SVMP_PIII}	{CTL}	0.619048	0.928571	1.392857	13
[438]	{CRiSP,CTL,Dis,SVSP}	{SVMP_PIII}	0.619048	1	1.05	13
[439]	{CRiSP,CTL,SVMP_PIII,SVSP}	{Dis}	0.619048	1	1.235294	13
[440]	{CRiSP,CTL,Dis,SVMP_PIII}	{SVSP}	0.619048	1	1.05	13
[441]	{CTL,Dis,SVMP_PIII,SVSP}	{CRiSP}	0.619048	0.928571	1.3	13
[442]	{CRiSP,Dis,SVMP_PIII,SVSP}	{CTL}	0.619048	0.928571	1.392857	13
[443]	{CRiSP,CTL,Dis,SVSP}	{PLA2}	0.619048	1	1	13
[444]	{CRiSP,CTL,PLA2,SVSP}	{Dis}	0.619048	1	1.235294	13

[445]	{CRiSP,CTL,Dis,PLA2}	{SVSP}	0.619048	1	1.05	13
[446]	{CTL,Dis,PLA2,SVSP}	{CRiSP}	0.619048	0.928571	1.3	13
[447]	{CRiSP,Dis,PLA2,SVSP}	{CTL}	0.619048	0.928571	1.392857	13
[448]	{CRiSP,CTL,SVMP_PIII,SVSP}	{PLA2}	0.619048	1	1	13
[449]	{CRiSP,CTL,PLA2,SVSP}	{SVMP_PIII}	0.619048	1	1.05	13
[450]	{CRiSP,CTL,PLA2,SVMP_PIII}	{SVSP}	0.619048	1	1.05	13
[451]	{CTL,PLA2,SVMP_PIII,SVSP}	{CRiSP}	0.619048	0.928571	1.3	13
[452]	{CRiSP,PLA2,SVMP_PIII,SVSP}	{CTL}	0.619048	0.928571	1.392857	13
[453]	{CRiSP,CTL,Dis,SVMP_PIII}	{PLA2}	0.619048	1	1	13
[454]	{CRiSP,CTL,Dis,PLA2}	{SVMP_PIII}	0.619048	1	1.05	13
[455]	{CRiSP,CTL,PLA2,SVMP_PIII}	{Dis}	0.619048	1	1.235294	13
[456]	{CTL,Dis,PLA2,SVMP_PIII}	{CRiSP}	0.619048	0.928571	1.3	13
[457]	{CTL,Dis,LAAO,SVSP}	{SVMP_PIII}	0.666667	1	1.05	14
[458]	{CTL,LAAO,SVMP_PIII,SVSP}	{Dis}	0.666667	1	1.235294	14
[459]	{CTL,Dis,LAAO,SVMP_PIII}	{SVSP}	0.666667	1	1.05	14
[460]	{CTL,Dis,SVMP_PIII,SVSP}	{LAAO}	0.666667	1	1.235294	14
[461]	{Dis,LAAO,SVMP_PIII,SVSP}	{CTL}	0.666667	0.933333	1.4	14
[462]	{CTL,Dis,LAAO,SVSP}	{PLA2}	0.666667	1	1	14
[463]	{CTL,LAAO,PLA2,SVSP}	{Dis}	0.666667	1	1.235294	14
[464]	{CTL,Dis,LAAO,PLA2}	{SVSP}	0.666667	1	1.05	14
[465]	{CTL,Dis,PLA2,SVSP}	{LAAO}	0.666667	1	1.235294	14
[466]	{Dis,LAAO,PLA2,SVSP}	{CTL}	0.666667	0.933333	1.4	14
[467]	{CTL,LAAO,SVMP_PIII,SVSP}	{PLA2}	0.666667	1	1	14
[468]	{CTL,LAAO,PLA2,SVSP}	{SVMP_PIII}	0.666667	1	1.05	14
[469]	{CTL,LAAO,PLA2,SVMP_PIII}	{SVSP}	0.666667	1	1.05	14
[470]	{CTL,PLA2,SVMP_PIII,SVSP}	{LAAO}	0.666667	1	1.235294	14
[471]	{LAAO,PLA2,SVMP_PIII,SVSP}	{CTL}	0.666667	0.933333	1.4	14
[472]	{CTL,Dis,LAAO,SVMP_PIII}	{PLA2}	0.666667	1	1	14
[473]	{CTL,Dis,LAAO,PLA2}	{SVMP_PIII}	0.666667	1	1.05	14
[474]	{CTL,LAAO,PLA2,SVMP_PIII}	{Dis}	0.666667	1	1.235294	14

[475]	{CTL,Dis,PLA2,SVMP_PIII}	{LAAO}	0.666667	1	1.235294	14
[476]	{CTL,Dis,SVMP_PIII,SVSP}	{PLA2}	0.666667	1	1	14
[477]	{CTL,Dis,PLA2,SVSP}	{SVMP_PIII}	0.666667	1	1.05	14
[478]	{CTL,PLA2,SVMP_PIII,SVSP}	{Dis}	0.666667	1	1.235294	14
[479]	{CTL,Dis,PLA2,SVMP_PIII}	{SVSP}	0.666667	1	1.05	14
[480]	{CRiSP,Dis,LAAO,SVSP}	{SVMP_PIII}	0.619048	1	1.05	13
[481]	{CRiSP,LAAO,SVMP_PIII,SVSP}	{Dis}	0.619048	1	1.235294	13
[482]	{CRiSP,Dis,LAAO,SVMP_PIII}	{SVSP}	0.619048	0.928571	0.975	13
[483]	{CRiSP,Dis,SVMP_PIII,SVSP}	{LAAO}	0.619048	0.928571	1.147059	13
[484]	{CRiSP,Dis,LAAO,SVSP}	{PLA2}	0.619048	1	1	13
[485]	{CRiSP,LAAO,PLA2,SVSP}	{Dis}	0.619048	1	1.235294	13
[486]	{CRiSP,Dis,LAAO,PLA2}	{SVSP}	0.619048	0.928571	0.975	13
[487]	{CRiSP,Dis,PLA2,SVSP}	{LAAO}	0.619048	0.928571	1.147059	13
[488]	{CRiSP,LAAO,SVMP_PIII,SVSP}	{PLA2}	0.619048	1	1	13
[489]	{CRiSP,LAAO,PLA2,SVSP}	{SVMP_PIII}	0.619048	1	1.05	13
[490]	{CRiSP,LAAO,PLA2,SVMP_PIII}	{SVSP}	0.619048	0.928571	0.975	13
[491]	{CRiSP,PLA2,SVMP_PIII,SVSP}	{LAAO}	0.619048	0.928571	1.147059	13
[492]	{CRiSP,Dis,LAAO,SVMP_PIII}	{PLA2}	0.666667	1	1	14
[493]	{CRiSP,Dis,LAAO,PLA2}	{SVMP_PIII}	0.666667	1	1.05	14
[494]	{CRiSP,LAAO,PLA2,SVMP_PIII}	{Dis}	0.666667	1	1.235294	14
[495]	{CRiSP,Dis,PLA2,SVMP_PIII}	{LAAO}	0.666667	0.933333	1.152941	14
[496]	{CRiSP,Dis,SVMP_PIII,SVSP}	{PLA2}	0.666667	1	1	14
[497]	{CRiSP,Dis,PLA2,SVSP}	{SVMP_PIII}	0.666667	1	1.05	14
[498]	{CRiSP,PLA2,SVMP_PIII,SVSP}	{Dis}	0.666667	1	1.235294	14
[499]	{CRiSP,Dis,PLA2,SVMP_PIII}	{SVSP}	0.666667	0.933333	0.98	14
[500]	{Dis,LAAO,SVMP_PIII,SVSP}	{PLA2}	0.714286	1	1	15
[501]	{Dis,LAAO,PLA2,SVSP}	{SVMP_PIII}	0.714286	1	1.05	15
[502]	{LAAO,PLA2,SVMP_PIII,SVSP}	{Dis}	0.714286	1	1.235294	15
[503]	{Dis,LAAO,PLA2,SVMP_PIII}	{SVSP}	0.714286	0.9375	0.984375	15
[504]	{Dis,PLA2,SVMP_PIII,SVSP}	{LAAO}	0.714286	0.9375	1.158088	15

[505]	{Dis,LAAO,SVMP_PI,SVMP_PIII,SVSP}	{PLA2}	0.52381	1	1	11
[506]	{Dis,LAAO,PLA2,SVMP_PI,SVSP}	{SVMP_PIII}	0.52381	1	1.05	11
[507]	{LAAO,PLA2,SVMP_PI,SVMP_PIII,SVSP}	{Dis}	0.52381	1	1.235294	11
[508]	{Dis,LAAO,PLA2,SVMP_PI,SVMP_PIII}	{SVSP}	0.52381	0.916667	0.9625	11
[509]	{Dis,PLA2,SVMP_PI,SVMP_PIII,SVSP}	{LAAO}	0.52381	1	1.235294	11
[510]	{BPP,CRiSP,Dis,LAAO,SVMP_PIII}	{PLA2}	0.52381	1	1	11
[511]	{BPP,CRiSP,Dis,LAAO,PLA2}	{SVMP_PIII}	0.52381	1	1.05	11
[512]	{BPP,CRiSP,LAAO,PLA2,SVMP_PIII}	{Dis}	0.52381	1	1.235294	11
[513]	{BPP,CRiSP,Dis,PLA2,SVMP_PIII}	{LAAO}	0.52381	1	1.235294	11
[514]	{BPP,Dis,LAAO,PLA2,SVMP_PIII}	{CRiSP}	0.52381	1	1.4	11
[515]	{CRiSP,CTL,Dis,LAAO,SVSP}	{SVMP_PIII}	0.619048	1	1.05	13
[516]	{CRiSP,CTL,LAAO,SVMP_PIII,SVSP}	{Dis}	0.619048	1	1.235294	13
[517]	{CRiSP,CTL,Dis,LAAO,SVMP_PIII}	{SVSP}	0.619048	1	1.05	13
[518]	{CRiSP,CTL,Dis,SVMP_PIII,SVSP}	{LAAO}	0.619048	1	1.235294	13
[519]	{CTL,Dis,LAAO,SVMP_PIII,SVSP}	{CRiSP}	0.619048	0.928571	1.3	13
[520]	{CRiSP,Dis,LAAO,SVMP_PIII,SVSP}	{CTL}	0.619048	1	1.5	13
[521]	{CRiSP,CTL,Dis,LAAO,SVSP}	{PLA2}	0.619048	1	1	13
[522]	{CRiSP,CTL,LAAO,PLA2,SVSP}	{Dis}	0.619048	1	1.235294	13
[523]	{CRiSP,CTL,Dis,LAAO,PLA2}	{SVSP}	0.619048	1	1.05	13
[524]	{CRiSP,CTL,Dis,PLA2,SVSP}	{LAAO}	0.619048	1	1.235294	13
[525]	{CTL,Dis,LAAO,PLA2,SVSP}	{CRiSP}	0.619048	0.928571	1.3	13
[526]	{CRiSP,Dis,LAAO,PLA2,SVSP}	{CTL}	0.619048	1	1.5	13
[527]	{CRiSP,CTL,LAAO,SVMP_PIII,SVSP}	{PLA2}	0.619048	1	1	13
[528]	{CRiSP,CTL,LAAO,PLA2,SVSP}	{SVMP_PIII}	0.619048	1	1.05	13
[529]	{CRiSP,CTL,LAAO,PLA2,SVMP_PIII}	{SVSP}	0.619048	1	1.05	13
[530]	{CRiSP,CTL,PLA2,SVMP_PIII,SVSP}	{LAAO}	0.619048	1	1.235294	13
[531]	{CTL,LAAO,PLA2,SVMP_PIII,SVSP}	{CRiSP}	0.619048	0.928571	1.3	13
[532]	{CRiSP,LAAO,PLA2,SVMP_PIII,SVSP}	{CTL}	0.619048	1	1.5	13
[533]	{CRiSP,CTL,Dis,LAAO,SVMP_PIII}	{PLA2}	0.619048	1	1	13
[534]	{CRiSP,CTL,Dis,LAAO,PLA2}	{SVMP_PIII}	0.619048	1	1.05	13

[535]	{CRiSP,CTL,LAAO,PLA2,SVMP_PIII}	{Dis}	0.619048	1	1.235294	13
[536]	{CRiSP,CTL,Dis,PLA2,SVMP_PIII}	{LAAO}	0.619048	1	1.235294	13
[537]	{CTL,Dis,LAAO,PLA2,SVMP_PIII}	{CRiSP}	0.619048	0.928571	1.3	13
[538]	{CRiSP,Dis,LAAO,PLA2,SVMP_PIII}	{CTL}	0.619048	0.928571	1.392857	13
[539]	{CRiSP,CTL,Dis,SVMP_PIII,SVSP}	{PLA2}	0.619048	1	1	13
[540]	{CRiSP,CTL,Dis,PLA2,SVSP}	{SVMP_PIII}	0.619048	1	1.05	13
[541]	{CRiSP,CTL,PLA2,SVMP_PIII,SVSP}	{Dis}	0.619048	1	1.235294	13
[542]	{CRiSP,CTL,Dis,PLA2,SVMP_PIII}	{SVSP}	0.619048	1	1.05	13
[543]	{CTL,Dis,PLA2,SVMP_PIII,SVSP}	{CRiSP}	0.619048	0.928571	1.3	13
[544]	{CRiSP,Dis,PLA2,SVMP_PIII,SVSP}	{CTL}	0.619048	0.928571	1.392857	13
[545]	{CTL,Dis,LAAO,SVMP_PIII,SVSP}	{PLA2}	0.666667	1	1	14
[546]	{CTL,Dis,LAAO,PLA2,SVSP}	{SVMP_PIII}	0.666667	1	1.05	14
[547]	{CTL,LAAO,PLA2,SVMP_PIII,SVSP}	{Dis}	0.666667	1	1.235294	14
[548]	{CTL,Dis,LAAO,PLA2,SVMP_PIII}	{SVSP}	0.666667	1	1.05	14
[549]	{CTL,Dis,PLA2,SVMP_PIII,SVSP}	{LAAO}	0.666667	1	1.235294	14
[550]	{Dis,LAAO,PLA2,SVMP_PIII,SVSP}	{CTL}	0.666667	0.933333	1.4	14
[551]	{CRiSP,Dis,LAAO,SVMP_PIII,SVSP}	{PLA2}	0.619048	1	1	13
[552]	{CRiSP,Dis,LAAO,PLA2,SVSP}	{SVMP_PIII}	0.619048	1	1.05	13
[553]	{CRiSP,LAAO,PLA2,SVMP_PIII,SVSP}	{Dis}	0.619048	1	1.235294	13
[554]	{CRiSP,Dis,LAAO,PLA2,SVMP_PIII}	{SVSP}	0.619048	0.928571	0.975	13
[555]	{CRiSP,Dis,PLA2,SVMP_PIII,SVSP}	{LAAO}	0.619048	0.928571	1.147059	13
[556]	{CRiSP,CTL,Dis,LAAO,SVMP_PIII,SVSP}	{PLA2}	0.619048	1	1	13
[557]	{CRiSP,CTL,Dis,LAAO,PLA2,SVSP}	{SVMP_PIII}	0.619048	1	1.05	13
[558]	{CRiSP,CTL,LAAO,PLA2,SVMP_PIII,SVSP}	{Dis}	0.619048	1	1.235294	13
[559]	{CRiSP,CTL,Dis,LAAO,PLA2,SVMP_PIII}	{SVSP}	0.619048	1	1.05	13
[560]	{CRiSP,CTL,Dis,PLA2,SVMP_PIII,SVSP}	{LAAO}	0.619048	1	1.235294	13
[561]	{CTL,Dis,LAAO,PLA2,SVMP_PIII,SVSP}	{CRiSP}	0.619048	0.928571	1.3	13
[562]	{CRiSP,Dis,LAAO,PLA2,SVMP_PIII,SVSP}	{CTL}	0.619048	1	1.5	13

Table S2. Depictions of all association rules between proteins expressed in *Crotalus* venom using relative abundance data.

Rule No.	Protein Predictor		Protein Predicted	Support	confidence	lift
[1]	{SVMP_PI}	=>	{LAAO}	0.6	1	1.5
[2]	{BPP, CriSP}	=>	{LAAO}	0.6	1	1.5
[3]	{CriSP, CTL}	=>	{LAAO}	0.67	1	1.5
[4]	{LAAO}	=>	{SVMP_PI}	0.6	0.9	1.5
[5]	{CriSP, CTL}	=>	{SVMP_PI}	0.6	0.9	1.5
[6]	{CTL}	=>	{BPP}	0.67	0.91	1.36
[7]	{CTL}	=>	{LAAO}	0.67	0.91	1.36
[8]	{CriSP}	=>	{LAAO}	0.67	0.91	1.36
[9]	{SVMP_PI}	=>	{CTL}	0.6	1	1.36
[10]	{SVMP_PI}	=>	{CriSP}	0.6	1	1.36
[11]	{BPP}	=>	{CTL}	0.67	1	1.36
[12]	{LAAO}	=>	{CTL}	0.67	1	1.36
[13]	{LAAO}	=>	{CriSP}	0.67	1	1.36
[14]	{SVMP_PII, SVMP_PIII}	=>	{CTL}	0.53	1	1.36
[15]	{CTL, SVMP_PII}	=>	{SVMP_PIII}	0.53	1	1.36
[16]	{SVMP_PII, SVSP}	=>	{SVMP_PIII}	0.53	1	1.36
[17]	{SVMP_PII, SVSP}	=>	{CTL}	0.53	1	1.36
[18]	{BPP}	=>	{LAAO}	0.6	0.9	1.35
[19]	{LAAO}	=>	{BPP}	0.6	0.9	1.35
[20]	{PLA2 Other, SVSP}	=>	{SVMP_PIII}	0.73	0.92	1.25
[21]	{PLA2 Other, SVSP}	=>	{CTL}	0.73	0.92	1.25
[22]	{PLA2 Other, SVSP}	=>	{CriSP}	0.73	0.92	1.25
[23]	{SVMP_PIII}	=>	{CTL}	0.67	0.91	1.24
[24]	{CTL}	=>	{SVMP_PIII}	0.67	0.91	1.24
[25]	{SVMP_PIII}	=>	{CriSP}	0.67	0.91	1.24
[26]	{CriSP}	=>	{SVMP_PIII}	0.67	0.91	1.24
[27]	{CTL}	=>	{CriSP}	0.67	0.91	1.24

[28]	{CriSP}	=>	{CTL}	0.67	0.91	1.24
[29]	{BPP}	=>	{SVMP_PIII}	0.6	0.9	1.23
[30]	{BPP}	=>	{CriSP}	0.6	0.9	1.23
[31]	{LAAO}	=>	{SVMP_PIII}	0.6	0.9	1.23
[32]	{SVMP_PII}	=>	{PLA2 Other}	0.6	1	1.15
[33]	{MYO}	=>	{SVSP}	0.53	1	1.15
[34]	{SVMP_PI}	=>	{SVSP}	0.6	1	1.15
[35]	{SVMP_PI}	=>	{PLA2 Other}	0.6	1	1.15
[36]	{BPP}	=>	{SVSP}	0.67	1	1.15
[37]	{BPP}	=>	{PLA2 Other}	0.67	1	1.15
[38]	{SVMP_PIII}	=>	{SVSP}	0.73	1	1.15
[39]	{SVMP_PIII}	=>	{PLA2 Other}	0.73	1	1.15
[40]	{LAAO}	=>	{SVSP}	0.67	1	1.15
[41]	{LAAO}	=>	{PLA2 Other}	0.67	1	1.15
[42]	{CTL}	=>	{SVSP}	0.73	1	1.15
[43]	{CTL}	=>	{PLA2 Other}	0.73	1	1.15
[44]	{CriSP}	=>	{SVSP}	0.73	1	1.15
[45]	{CriSP}	=>	{PLA2 Other}	0.73	1	1.15
[46]	{SVSP}	=>	{PLA2 Other}	0.8	0.92	1.07
[47]	{PLA2 Other}	=>	{SVSP}	0.8	0.92	1.07
